# 3D extracellular matrix microenvironment in bioengineered tissue models of primary pediatric and adult brain tumors

**DOI:** 10.1038/s41467-019-12420-1

**Published:** 2019-10-04

**Authors:** Disha Sood, Min Tang-Schomer, Dimitra Pouli, Craig Mizzoni, Nicole Raia, Albert Tai, Knarik Arkun, Julian Wu, Lauren D. Black, Bjorn Scheffler, Irene Georgakoudi, Dennis A. Steindler, David L. Kaplan

**Affiliations:** 1 Department of Biomedical Engineering, Tufts University, Medford, MA 02155 USA; 2 Jackson Laboratory for Genomic Medicine, Farmington, CT 06032 USA; 3 Connecticut Children’s Medical Center, Harford, CT 06106 USA; 4 Department of Pathology, Beth Israel Deaconess Medical Center and Harvard Medical School, Boston, MA 02114 USA; 5 Genomics Core, Tufts University School of Medicine, Boston, MA 02111 USA; 6 Department of Pathology and Laboratory Medicine, Tufts Medical Center, Boston, MA 02111 USA; 7 Department of Neurosurgery, Tufts Medical Center, Boston, MA 02111 USA; 8 Department of Neuroscience, University of Florida, McKnight Brain Institute, Gainesville, FL 32610 USA; 9 DKFZ-Division of Translational Oncology/ Neurooncology, German Cancer Consortium (DKTK), Heidelberg & University Hospital Essen, Essen, Germany; 10 Neuroscience and Aging Laboratory, Jean Mayer USDA Human Nutrition Research Center on Aging, Tufts University, Boston, MA 02111 USA

**Keywords:** Tissue engineering, Cancer in the nervous system, Biomedical engineering

## Abstract

Dynamic alterations in the unique brain extracellular matrix (ECM) are involved in malignant brain tumors. Yet studies of brain ECM roles in tumor cell behavior have been difficult due to lack of access to the human brain. We present a tunable 3D bioengineered brain tissue platform by integrating microenvironmental cues of native brain-derived ECMs and live imaging to systematically evaluate patient-derived brain tumor responses. Using pediatric ependymoma and adult glioblastoma as examples, the 3D brain ECM-containing microenvironment with a balance of cell-cell and cell-matrix interactions supports distinctive phenotypes associated with tumor type-specific and ECM-dependent patterns in the tumor cells’ transcriptomic and release profiles. Label-free metabolic imaging of the composite model structure identifies metabolically distinct sub-populations within a tumor type and captures extracellular lipid-containing droplets with potential implications in drug response. The versatile bioengineered 3D tumor tissue system sets the stage for mechanistic studies deciphering microenvironmental role in brain tumor progression.

## Abstract (web summary)

The brain extracellular matrix (ECM) is altered in brain tumors, but its role in cancer progression and drug sensitivity are difficult to study. Here the authors develop a 3D bioengineered brain tissue model using patient-derived samples and tunable brain-derived ECM to examine the interplay between cells and the ECM.

## Introduction

Many brain tumors have dismal prognosis, especially glioblastoma (GBM) and those that typically occur in young children, such as ependymoma. Genome-wide profiling of these tumors have revealed increasing tumor subtypes and intra-tumor heterogeneities^1,2^. For example, it is found that brain tumor types are highly dependent on a patient’s age and brain region^3^; brain region/age-matched xenograft mouse models can better recapitulate the tumor cell biology than grafts in non-brain microenvironment^4^. Although brain-ECM receptors/ligands are increasingly been characterized^5–7^; brain-ECM studies in general have been difficult, in part, due to lack of access to specific matrix components for in vitro testing. We have attempted to develop an in vitro platform system to examine the dynamic interactions of a brain-ECM-containing microenvironment with different primary brain tumors. GBM, the most prevalent primary infiltrating glioma in adults, has a dismal prognosis with a median survival of 1–2 years post diagnosis^8^. Anaplastic ependymoma, a rapidly growing glioma arising from ependymal cells lining the ventricles, is among the three most common malignant pediatric brain tumors^9^. The two distinctive tumor types share little in common with regards to their biology other than the brain origin; therefore allowing for the distinction of tumor-specific vs. ECM-specific responses under the same ECM microenvironment.

ECM guides gene expression patterns within cells by influencing the cytoskeletal geometry and the chromatin-nuclear matrix interactions, and in turn cells impact the ECM, as established by the ‘dynamic reciprocity’ model^10^. The instructive role of basement membrane hydrogel microenvironment in directing epithelial tissue-specific responses has been well described in the context of mammary gland and breast tumorigenesis^10,11^. In comparison, few studies have directly examined the dynamic changes of brain ECM during development and in disease states^7^. The brain has a unique ECM composition with proteoglycans as a major component, including chondroitin sulfate proteoglycans (CSPGs), levels of which change during development^7^. Alterations in brain ECM associated with brain tumors include abnormally high levels of hyaluronan (HA) and/or collagens^12,13^ and altered expression of specific CSPGs^14^. While several CSPGs are upregulated in GBM progression^7^, CSPGs may also prevent diffuse invasion of tumor cells^15^. These studies have provided evidence of brain-ECM involvement in tumor progression; yet it is unclear how different tumors alter their ECM environment by the tumor cells’ reciprocal signaling. In order to understand the dynamic cell-ECM interactions, it is critical to separately define the contributions of ECM components towards brain tumor development, progression, and tumor-type-specific responses. In our in vitro platform system, we incorporate developmentally sourced brain-ECM cues, with the aim of capturing some of these dynamic tumor cell-ECM responses.

Current systems for investigating brain tumor biology include 2D monolayer cultures, neurosphere/organotypic cultures and animal models; each of which have limitations. Conventional 2D cell cultures lack a 3D ECM environment that is necessary for studying tumor cell-microenvironment interactions, while commonly used animal models are cost intensive, low-throughput, and difficult to translate to humans^16,17^. Additionally, existing 3D brain tumor models have primarily focused on mechanical effects of the matrix towards tumor growth and have relied on established tumor cell lines such as U87, U-251 MG^16,18,19^, which lack the cellular and structural heterogeneity present in an in vivo tumor. Also, despite in vivo malignancy, in vitro culture has not been successful with many cell lines^20^, making the need for brain-mimicking model systems particularly acute. Some patient-derived 3D tumor models, such as spheroids/organoids have been developed; however, they lack brain-specific ECM, are challenging to culture long-term due to expanding necrotic cores and a noticeable decline in growth, and have limited tunability^17,21^. Therefore, a brain-mimicking tumor model requires the combination of genuine tumor cells and brain-ECM-containing microenvironment.

Towards that end, we present a tunable platform to systematically examine the interactions of 3D brain-ECM-containing microenvironment with patient-derived brain tumor cells. We previously developed a 3D bioengineered brain tissue model based on a donut-shaped silk fibroin protein scaffold infused with ECM hydrogels. The compartmentalized model design permits region-specific primary neuronal growth in the soft hydrogel region vs. the stiffer scaffold outer-ring region^22^. In the current study, we leveraged the modular assembly for investigation of different ECM components; for example, supplement of fetal or adult porcine brain-derived ECM in a base hydrogel towards primary tumor growth/migration. Additionally, we integrated high resolution two-photon metabolic imaging into the bioengineered tissue model for real-time tracking of patient-derived tumor growth in 3D. Quantitative readouts of metabolic activity at cellular/subcellular features over several hundred microns of depth can be extracted from analysis of two-photon excited fluorescence (TPEF) images that rely on the natural fluorescence of two key metabolic co-enzymes, NADH (Nicotinamide adenine dinucleotide) and FAD (Flavin adenine dinucleotide) without the need for tissue processing or interference with cell viability or function^23–25^. The ability of the optical redox ratio to report changes in the redox state of cells has been validated with mass spectrometry measurements and its sensitivity to metabolic perturbations has been demonstrated^24,25^. For example, a high redox ratio (FAD/FAD + NADH) has been associated with enhanced glutaminolysis, uncoupling, and oxidative stress/cell apoptosis while a lower redox ratio with upregulated glycolysis and fatty acid oxidation/synthesis^23–26^.

**Fig. 1:**
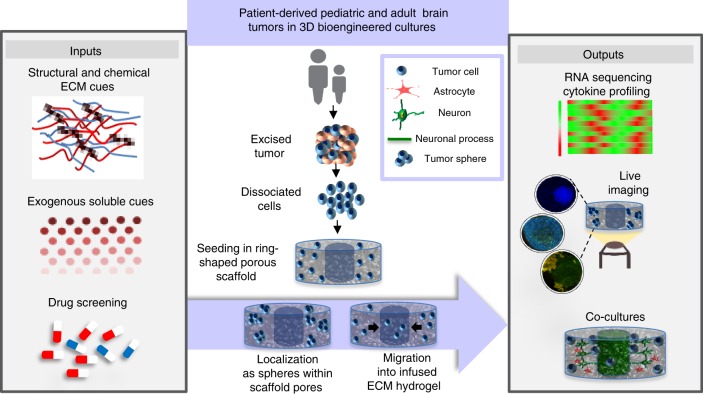
Versatile 3D bioengineered brain tumor tissue model. Multi-level interrogation into the role of extracellular matrix and microenvironment on tumor growth. Middle panel—Tumor cells seeded as single cells post dissociation from the excised tumor, migrate as single cells towards the central hydrogel window or revert back to sphere-like morphology within the scaffold depending on original tumor characteristics. Left panel—Introduction of suitable ECM and soluble cues to maintain tumor characteristics and drug screening. Right panel—Multiscale interrogation of the culture system via transcriptomic and genomic profiling, and live metabolic imaging. Co-culture of tumor cells with healthy differentiated human neural stem cells (hNSCs)

Here, we establish utility of the generated 3D bioengineered brain tumor system using pediatric anaplastic ependymoma and adult GBM patient-derived tumor cells as case studies. Tumor growth and drug responses in different microenvironments were assessed longitudinally using TPEF imaging, high throughput matrix metalloproteinases (MMPs)/cytokine release microarrays, and transcriptomic profiling (Fig. 1). We present a versatile brain tumor culture system with a unique advantage to tune the ECM microenvironment and simultaneously monitor the reciprocal signaling with the tumor cells. Our findings of tumor-type-specific ECM response demonstrate the potential for future mechanistic studies along with co-culture of tumor cells with healthy brain cells (Fig. 1). Thus, this platform, combining bioengineered scaffolds to provide high surface area to volume 3D environment for tumor cells and live imaging approaches, can offer opportunities for comprehensive understanding of the brain tumor-ECM microenvironment.

## Results

### Patient tumors displayed ECM-dependent metabolic activity

We sought to decipher the role of brain-derived ECM from varying developmental stages on brain tumor progression in vitro, to determine more optimal matrix conditions than existing methods for primary tumor culture. A decellularization approach was undertaken since little is known about brain ECM during development and ECM proteins are mostly conserved over species, such that porcine brains can be used to extract a mixture of native brain-ECM^27^. Additionally, in this study we employ a 3D donut-shaped scaffold-based approach to allow for simultaneous maintenance of both non-migratory and adherent migratory cell populations.

**Fig. 2:**
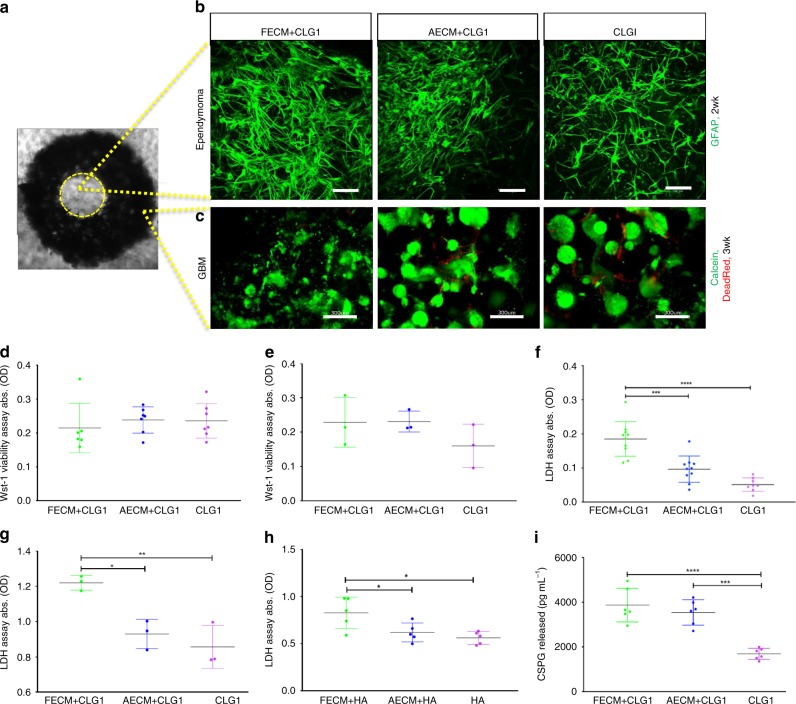
Patient-derived anaplastic ependymoma and glioblastoma in silk scaffold-based 3D constructs. **a** Brightfield image of donut-shaped silk scaffold seeded with tumor cells and infused with hydrogel. Systems infused with either collagen I (CLG1) or hyaluronic acid (HA) hydrogels supplemented with porcine brain-derived ECM (Fetal ECM: FECM, Adult ECM: AECM), cultured in chemically defined media lacking FBS. The middle circular window indicated by the yellow outline demarcates a distinct region within the construct that is exclusively hydrogel filled and not initially seeded with cells but where tumor cells can migrate. **b** Migration of anaplastic ependymoma cells at 2 week shown by GFAP staining within the middle hydrogel window of the 3D donut-shaped constructs. Presence of GFAP-positive glial cells matched histopathology reports. Scale bar, 100 μm, Max projection of z-stack imaged in Leica SP8 confocal. **c** Growth of glioblastoma (GBM) at 3 week shown by live calcein and dead red staining within the ring portion of the 3D donut-shaped constructs. GBM grew more as spheres and did not infiltrate the middle window at earlier time points in CLG1-based hydrogels. Scale bar, 300 μm, Max projection of z-stack imaged in Leica SP8 confocal. Wst-1 viability assay at 2 week in 3D ependymoma **d** and 1.5 mo GBM **e** silk-CLG1 cultures, respectively indicating similar viability across all ECM conditions. **f** Lactate dehydrogenase (LDH) release assay at 2 week in 3D ependymoma cultures. Statistically significant difference in the LDH release in FECM condition in comparison to AECM or CLG1 alone. **g** LDH release assay at 1.5 mo in 3D GBM silk-CLG1 cultures, showing statistically significant (higher) LDH release in FECM condition assuming a Gaussian distribution. **h** LDH release assay in 3D GBM silk-HA cultures, showing statistically significant (higher) LDH release in FECM condition at 1 mo. **i** Chondroitin sulfate proteoglycan (CSPG) release observed to be statistically significant (higher) in the FECM and AECM containing constructs at 3 week in 3D ependymoma cultures. Ordinary one-way ANOVA with Tukey’s post-hoc, F > 6.958, *p < 0.0412, **p = 0.0056, ***p = 0.0001, ****p < 0.0001. Error bars indicate mean ± s.d, Source data are provided as a Source Data file

Pediatric and adult patient-derived anaplastic ependymoma and GBM, respectively, were cultured in silk scaffold-based 3D constructs infused with either collagen type I (CLG1, shown to be compatible with brain cells^28^) or hyaluronic acid (HA, a defined brain-ECM component) hydrogels supplemented with native brain-derived ECM. Figure 2a, b shows infiltration towards the central hydrogel window of the 3D construct by GFAP-stained ependymoma cells. Ependymoma cells migrated as single cells into the hydrogel window, with faster migration qualitatively observed in the fetal ECM-enriched constructs (Fig. 2a, b). GBM, on the other hand, grew primarily as spheres within the silk scaffold-hydrogel constructs along with visibly more disperse cells within the fetal ECM-enriched hydrogels (Fig. 2a, c, Supplementary Fig. 1); however, there was little to no migration of GBM cells within the central window of CLG1-based constructs at earlier time points. When the same GBM was cultured in HA-based hydrogels (known to be abnormally high in GBM^13^), greater migration within shorter time frames was evident in the HA hydrogels, particularly within the fetal ECM-enriched HA cultures, at 1 month post-gel addition (Supplementary Fig. 2). Spheres of fibrous tumor were seen to migrate outwards from the outer rim of the scaffold forming what we define as the outer edge gel population (Supplementary Fig. 2 inset). This outward migration pattern was not noticed in collagen I hydrogels. Multiple spheres of varying sizes were observed within the 3D constructs for GBM, as opposed to all the spheres joining into one large mass when cultured by conventional scaffold-free cultures, pointing towards a competition between cell-cell and cell-matrix interactions in the 3D constructs with relevance to in vivo scenarios.

We employed different assays to determine the possible metabolic activities underlying the tumor-type-specific phenotypes. Despite using two completely distinct brain tumors, the cultured ependymoma and GBM exhibited similar trends in their metabolic activities in response to the infused hydrogel matrices. Analogous bulk viability was observed across different ECM conditions for both ependymoma and GBM (Fig. 2d, e). However, significantly higher lactate dehydrogenase (LDH) release was noted in the fetal ECM-enriched constructs in comparison to other ECMs for both tumor types (Fig. 2f–h). Similarly, when different base hydrogels of CLG1 or HA were utilized, LDH release showed higher levels in fetal ECM-enriched compared to unsupplemented constructs (Fig. 2g, h). These results indicate that the presence of fetal ECM promotes tumor cell metabolism for GBM as well as ependymoma.

Because certain ECM components such as CSPGs are known to inhibit tumor infiltration^15^, we suspected that the phenotypic differences between GBM and ependymoma despite the same starting ECM may relate to the modulation of tumor-secreted matrix components. Therefore, we examined CSPG levels in different tumor cultures. The released CSPGs were significantly higher in the fetal ECM-enriched constructs for ependymoma (Fig. 2i), but no CSPGs were detected from cultured GBM under any conditions at tested time points (1week–7.5 months).

Together, these data showed that in the same starting bioengineered microenvironment, the different tumor-type-specific phenotypes (i.e., more migratory ependymoma vs. sphere GBM) may attribute to tumor-released signals that can be identified by various non-destructive solution-based assays. Because primary tumor cases are often unpredictable; the fact that these 3D cultures can be monitored with specific live assays allowed for individualized studies tailored for tumor-type/stages. Based on the robust growth in fetal ECM-containing condition for both tumor types, we focused on this condition and examined their transcriptomic and proteomic characteristics.

### Transcriptomic profile clustered based on culture conditions

RNA sequencing of the 3D bioengineered tumor cultures was performed to determine genome-wide expression (transcriptomic) changes under different 3D tissue model conditions within a particular tumor type. The goal was to track how the tumor culture changes in terms of gene expression pertaining to media conditions, ECM, and time in culture.

**Fig. 3:**
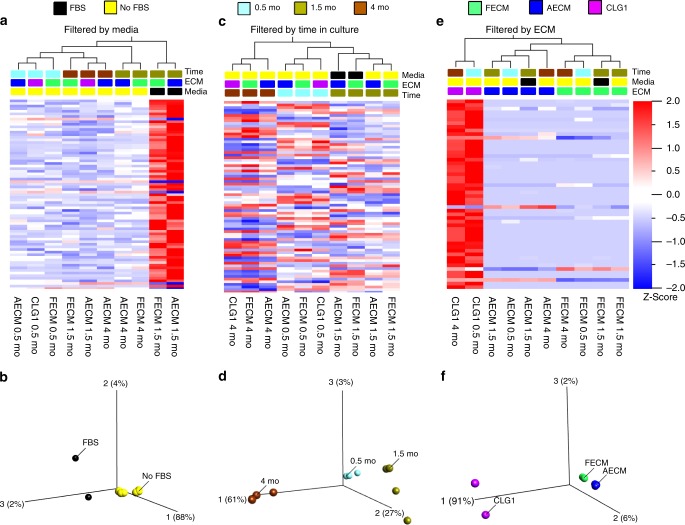
RNA-sequencing data for anaplastic ependymoma 3D bioengineered cultures. **a**–**c** Heatmap along with cluster dendrograms focusing on time in culture, media type and extracellular matrix categories, respectively, generated using FPKM (Reads Per Kilobase of transcript per Million mapped reads) values with a false discovery rate q < 0.2 or *p*-values < or = 0.005. **d**–**f** Principal component analysis (PCA) plots corresponding to **a**, **b** and **c** clustering, respectively. FECM- Fetal ECM + collagen I, AECM- Adult ECM + Collagen I, CLG1-Collagen I. **a**, **b**: False discovery rate (q) = 0.04, *p-*value = 0.001; **c**/**d**: q = 0.04, *p* = 0.005; **e**, **f**: q = 0.2, *p* = 0.0002; *n* = 1 per condition. Source data are provided as Supplementary Data 1

For ependymoma, clustering and principal component analysis (PCA) was performed across three experimental categories; media type (FBS vs. serum free), time in culture (0.5, 1.5, 4 months), and ECM (fetal brain-ECM-enriched collagen I- FECM, adult brain-ECM-enriched collagen I-AECM or plain collagen I-CLG1) (Fig. 3). When compared between media types, 3D tissue models in serum-free media showed more similar transcriptomic profiles (i.e., clustered together) when compared to those in serum-containing media. (Fig. 3a, b). We also noticed that primary pediatric ependymoma cells had difficulty thriving in FBS-containing media. On the other hand, when cultured in a chemically defined media generated in vitro cultures of ependymoma cells with in vivo-like rosette morphology^29^, increased metabolic activity and decreased drug response; consistent with recent reports of serum rendering enhanced drug sensitivity to tumor cells^30^ (Supplementary Fig. 3). Within the serum-free sub-group, fetal ECM cultures segregated away from the adult ECM and unsupplemented collagen conditions at both 0.5 and 4 months in culture (Fig. 3a). When compared between time points, the longest time point (4 months) samples showed different transcriptomic profiles than earlier time points (0.5 and 1.5 months) (Fig. 3c, d). Finally, when compared between ECM types, the unsupplemented collagen condition separated from fetal and adult ECM conditions, which overall had similar gene expression profiles regardless of time point or media type (Fig. 3e). The heatmap results of transcriptomic profiles were consistent with clustering on the PCA plots, where the distance between samples on the plot is proportional to the mean fold change of gene expression differences between samples (Fig. 3b, d, f). Specific genes consistent with studies of human ependymoma samples showed expression level changes^4^, including upregulated *insulin growth factor 2 (IGF2)*, *vascular endothelial growth factor-A (VEGFA), vimentin, matrix metalloproteinase 9 (MMP9) and MMP2*^31^ and downregulated mature neuronal maker, *microtubule associated protein 2 (MAP2)* in fetal ECM-enriched cultures when compared to unsupplemented collagen cultures (Supplementary Figs. 4, 5).

**Fig. 4:**
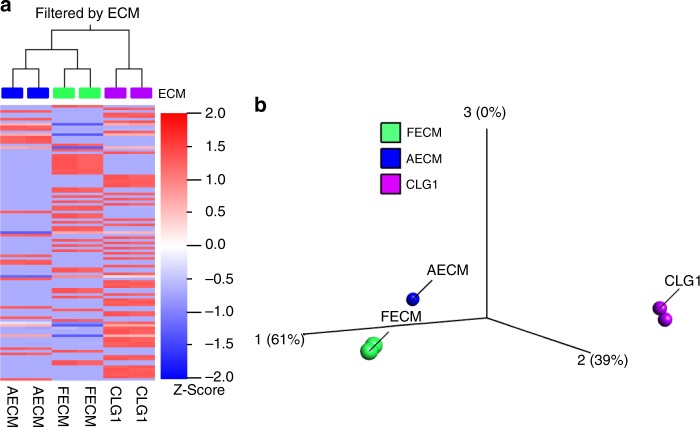
RNA-sequencing data for glioblastoma 3D bioengineered cultures. **a** Heatmap along with clustering based on extracellular matrix generated using FPKM (Reads Per Kilobase of transcript per Million mapped reads) values. **b** Principal component analysis (PCA) plot. FECM- Fetal ECM + collagen I, AECM- Adult ECM + Collagen I, CLG1-Collagen I. False discovery rate (q) = 0.04, *p-*value = 0.00008; *n* = 2 per condition Source data are provided as Supplementary Data 1

For the GBM cultures, fetal and adult ECM constructs also showed more similarity to each other than to the unsupplemented collagen I constructs, as seen in heat maps with dendrogram clustering alongside PCA plots (Fig. 4a, b). Fetal ECM-enriched cultures supported upregulation of genes found to be associated with GBM human samples including *epidermal growth factor receptor (EGFR)*^32^, *insulin growth factor binding protein 2 (IGFBP2)*^33^ and *MMP2*^34^, compared to other ECM conditions (Supplementary Fig. 6). We additionally validated higher expression of *EGFR* (~1.5 fold, *p* < 0.05) in fetal ECM-enriched GBM cultures relative to unsupplemented collagen I by PCR (Supplementary Fig. 7).

Together, these data showed that the 3D model’s culture conditions affect tumor tissue’s gene expression profiles, and suggest that these genetic changes underlie the phenotypic outcomes of tumor growth in the different microenvironments. In particular, 3D tissue models with brain-ECM-containing hydrogels (i.e., derived from fetal or adult brain sources) differed from those with non-brain-derived ECM hydrogels (i.e., unsupplemented collagen I) for both ependymoma and GBM.

### Tumor-type-specific interactions with ECM microenvironments

Cytokines released from the 3D bioengineered ependymoma and GBM cultures were examined to determine impact of the microenvironment on tumor-secreted factors and also tumor-type-specific paracrine signaling. We studied ECM-associated profiles including MMPs, tissue inhibitors of MMPs (TIMPs), and cytokines with culture supernatants of 3D tumor models.

**Fig. 5:**
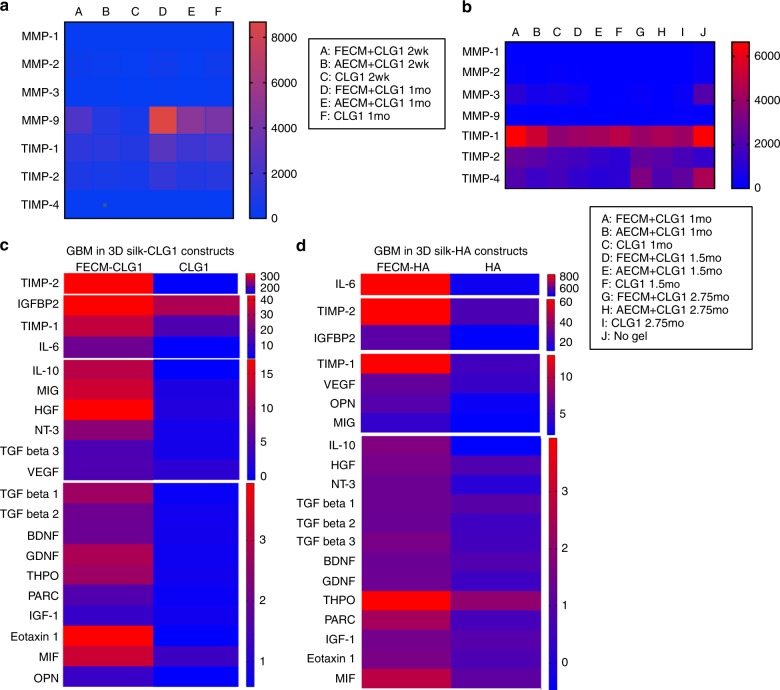
Cytokine and MMP/TIMP media profiling from 3D bioengineered cultures. **a** MMP/TIMP release profile (reported in pg mL^−1^) of 3D ependymoma cultures infused with CLG1-based hydrogels. *n* = 5 pooled samples per condition. **b** MMP/TIMP release profile (reported in pg mL^−1^) of 3D GBM cultures infused with CLG1-based hydrogels alongside 3D GBM constructs with no hydrogel added. *n* = 5 pooled samples per condition. **c** Cytokine release profile at 1 mo of 3D GBM cultures infused with CLG1-based hydrogels (reported as fold change over control media). *n* = 7 pooled samples per condition. **d** Cytokine release profile at 6.5 mo of 3D GBM cultures infused with HA-based hydrogels at 5.5 mo time point (reported as fold change over control media). *n* = 5 pooled samples per condition. Supplementary Table 1 lists the full forms of the cytokine abbreviations. Source data are provided as a Source Data file

With ependymoma tissue models, higher (2–4 fold) MMP-9 release was observed in cultures containing fetal ECM, while TIMPs were detected at much lower levels overall (0.3–0.5 fold) compared to other ECM types (Fig. 5a). This feature correlated with the high metabolic activity (LDH assay) and tissue viability (Fig. 2d, f), the highly migratory phenotype (Fig. 2b) and tumor-released CSPG (Fig. 2i) of this group.

With GBM tissue models, TIMPs were upregulated across all ECM conditions as opposed to MMPs (Fig. 5b, Supplementary Fig. 8a). In particular, TIMP2 was elevated in fetal ECM-enriched cultures (~2.5 fold) over unsupplemented CLG1 or HA cultures (Fig. 5c, d). When either CLG1 or HA was used as a base hydrogel in GBM cultures, fetal ECM supplement showed the upregulation of factors that are associated with tumor aggressiveness and growth, including IL-6, IGFBP2, MCP-1, TIMP-2, TIMP-1, MIG, IL-10, HGF, and NT-3^33–36^ (Fig. 5c, d, Supplementary Fig. 8b, c).

### Real-time tracking of 3D tumor growth with metabolic imaging

GBM is known to harbor metabolically heterogeneous cell populations that respond differentially to chemotherapy drugs^37^, which is imperative to recapitulate in in vitro models^38,39^. We sought to determine the presence of such populations in the 3D tumor models and their drug responses using high resolution metabolic imaging to dynamically monitor the tumor growth.

**Fig. 6:**
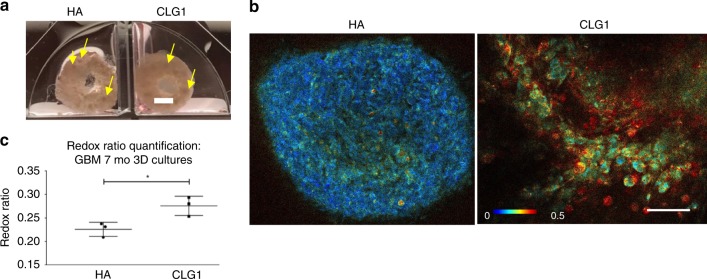
Live metabolic imaging of glioblastoma cultured in 3D silk-CLG1 or silk-HA constructs. **a** Macroscopic views of the GBM spheres within silk-CLG1 or silk-HA constructs. Scale bar, 2 mm. The yellow arrows point at spontaneously forming sphere-like structures from the seeded tumor cells post dissociation. **b** Representative redox ratio images from endogenous metabolic co-factors FAD and NADH within GBM cells in 3D constructs at 1.5 mo post-gel addition and 7 mo post cell seeding. Scale bar, 50 µm. Image heatmap reflects varying redox ratio intensities. **c** Quantitative intracellular signal evaluation validated the existence of metabolic differences between HA and CLG1 conditions, with a statistically significantly higher redox ratio (FAD/FAD + NADH) in CLG1 cultures. Unpaired two-tailed *t*-test, *p* = 0.0271, df = 4, *t* = 3.406, *n* = 3 per condition (Multiple areas imaged per condition = 3–8; total imaged in CLG1 = 13, HA = 19). Error bars indicate mean ± s.d, Source data are provided as a Source Data file

First, we compared GBM growth in HA vs. CLG1 infused cell-seeded scaffolds. Upon hydrogel introduction at 5.5 months, and post 1.5 month of hydrogel infusion, macroscopic spheres were visible in both HA and CLG1 conditions (Fig. 6a, yellow arrows). Endogenous imaging of metabolic enzymes indicated metabolic differences between HA and CLG1 conditions, with a significantly higher redox ratio (FAD/FAD + NADH) in CLG1 condition that is visually manifested as more intense red hues in the redox-scaled color-coded images and quantified by the optical redox ratio (Fig. 6b, c).

**Fig. 7:**
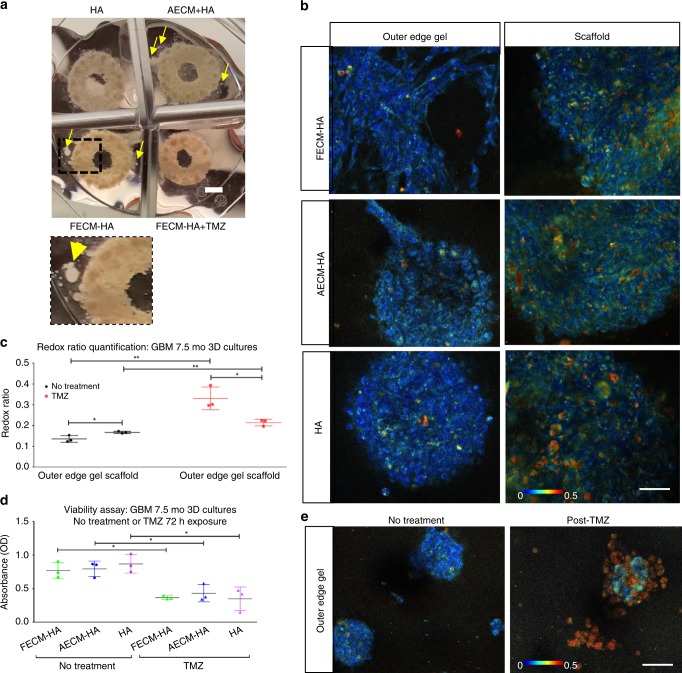
Live metabolic imaging of glioblastoma cultured in 3D silk-HA-ECM constructs. **a** Macroscopic views of the GBM spheres within silk-HA-ECM constructs, post- DMSO (no treatment control) or temozolomide (TMZ) exposure. Scale bar, 2 mm. **b** Representative redox ratio images from endogenous metabolic co-factors FAD and NADH within GBM cells in 3D silk-HA constructs at 2 mo post-gel addition and 7.5 mo post cell seeding, with right panel showing images taken within the scaffold portion and left panel with images taken in the outer edge gel of the construct, respectively. Scale bar, 50 µm. Image heatmap is the same for panel images and reflects varying redox ratio intensities. **c** Redox ratio quantification corresponding to outer edge gel areas and within scaffold areas, post-DMSO or TMZ exposure. Unpaired two-tailed *t*-tests between separate pairs; *n* = 3 per condition (multiple areas imaged per condition),**p < 0.0081, *p < 0.0321, df = 4, t > 3.228. **d** Wst-1 bulk viability assay at 7.5 mo in 3D GBM silk-HA cultures post- DMSO and TMZ exposure. Unpaired two-tailed *t*-test between separate pairs, *p < 0.0221, df = 4, t > 3.635. **e** Representative redox ratio images within GBM cells in 3D silk-HA constructs at 2 mo post-gel addition and 7.5 mo post cell seeding. 2-photon signals were obtained post- 72 h exposure to either DMSO or TMZ. Scale bar, 50 µm. Error bars indicate mean ± s.d, Source data are provided as a Source Data file

The HA condition was evaluated longitudinally alongside two additional conditions; fetal brain-ECM-enriched HA (FECM-HA) and adult brain-ECM-enriched HA (AECM-HA). Though tumor cells were only seeded in the ring-shaped silk scaffold; within the first 2 weeks post-gel addition, tumor cell growth was observed in the gel portion extending from the outer edges of the brain-ECM-enriched HA-containing silk scaffolds (noticed via daily inspections under bright-field) (Supplementary Fig. 2).We refer to this newly arisen population observed under bright-field (indicated by yellow arrows in Fig. 7a) and via metabolic imaging (Fig. 7b) as the outer edge gel cell population. Although similar in redox ratio hues, varied structural organization of the cellular populations was visible in the outer edge gel depending on the ECM supplementation. These cells showed sphere morphology in pure HA gels, while a greater tendency of cellular spreading was observed in brain-ECM-enriched HA gels, suggesting a locally infiltrative phenotype (Fig. 7b). The locally infiltrative cell populations (indicated by yellow arrows in Fig. 7a) had an overall lower redox ratio qualitatively observed by darker blue hues and quantified by the optical redox ratio (Fig. 7b, left column; Fig. 7c, black data points) in comparison to the immobile cell populations within the bulk of the scaffold region (Fig. 7b, right column; Fig. 7c, black data points), likely indicating a more proliferative/glycolytic phenotype.

Upon TMZ treatment (200 ug mL^−1^, 72 h), bulk wst-1 assay results at one-week post-treatment exhibited a significant decrease in viability across all HA conditions (Fig. 7d). Correspondingly, metabolic imaging showed a significant increase in redox ratio post drug treatment across all ECM conditions and of both the scaffold and outer edge gel cell populations (Fig. 7c). However, the cellular patterns observed in the redox ratio images revealed cell population-specific differences of metabolic activities; the outer edge cell population had concurrent loss in cell feature morphology and significantly increased redox ratio hues in comparison to TMZ treated scaffold populations (Fig. 7c, e). There was also significant cell loss with this population as observed macroscopically by not many visible cells within the outer gel across all conditions post TMZ treatment (seen during imaging when sampling outer edge gel areas) (Fig. 7a). This finding is supportive of the detected metabolic patterns of the outer edge gel populations, as more actively proliferating populations would be highly targeted by the DNA alkylating TMZ treatment. Similarly, the detection of undisturbed cellular clusters in our cultures hints towards TMZ-resistant populations, a finding matching clinical observations of high rates of TMZ patient non-responders^40^.

Detailed pixel-wise analysis of the redox ratio images further revealed four distinct redox distributions (defined as Components 1–4, with Component 1 and Component 4 centered at the lowest and highest redox ratios, respectively), where Components 3 and 4 had significantly greater relative weight with the cells in the outer edge gel, suggesting increased oxidative stress and apoptotic trends in this population post drug treatment^26^ (Supplementary Fig. 9). In addition, these cells with concurrent loss in cell feature morphology and increased redox ratio were confirmed to be indeed apoptotic by live caspase 3/7 positive staining (Supplementary Fig. 10). Together, these data indicate that the outer edge gel cell population was more drug-responsive to TMZ treatment than the immobile population in the scaffold region. Thus, metabolic imaging revealed tumor growth differences across ECM conditions (HA vs. CLGI base hydrogels), and varying response of metabolically heterogeneous cell populations post drug treatment. Such differences were not discernable by bulk viability assays.

### Lipid droplets observed in 3D bioengineered GBM cultures

**Fig. 8:**
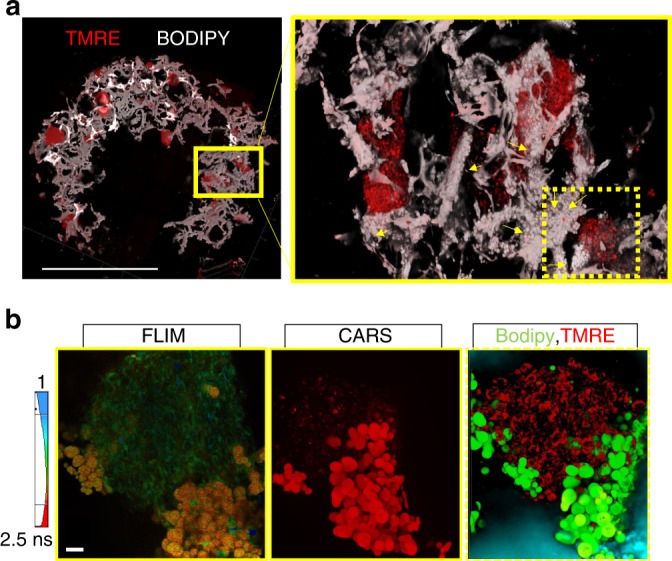
Lipid droplets in glioblastoma cultured in 3D silk-HA-ECM constructs. **a** Spread of individual GBM spheres of varying sizes as indicated by TMRE staining, within the silk-HA constructs and a zoomed in area in the right panel, where droplets are visible as indicated by the yellow arrows. The droplets are white spherical structures against silk scaffold, which is also seen in white corresponding to the fluorescence signal. Stitching and 3D rendering done to obtain the images. Scale bar, 2 mm. **b** Confirmation of the droplets visible within the silk-HA constructs to be lipid-containing, as indicated by an increased fluorescence lifetime in comparison to GBM cells captured by 2-photon lifetime imaging (leftmost panel, one plane), by CARS signal targeting the C–H stretch shown for droplets (bright red) and cells (faint red) (middle panel), and by BODIPY staining of lipid droplets (green) against GBM cells stained by TMRE (red) captured by confocal imaging. CARS and confocal images are presented as maximal projections of 3D z-stacks to optimize visual contrast. Scale bar, 20 µm

We observed the production of lipid-containing droplets within hydrogel infused 3D GBM in vitro cultures (Fig. 8a) during TPEF imaging. These droplets in comparison to adjacent GBM cells had a much higher fluorescence lifetime (Fig. 8b, left panel), which has previously been indicated for lipid droplets in adipose tissue^41^. Additionally, BODIPY dye that stains for neutral lipids was utilized, and Coherent Anti-stokes Raman Spectroscopy (CARS) was employed since it can delineate the morphochemical properties of all lipid droplets in unstained samples^42^. The lipid signature of the observed droplets was confirmed both by CARS targeting the C–H molecular vibrations (Supplementary Movie 1) and BODIPY staining (Fig. 8b, middle panel). There were few to no lipid droplets (LDs) visible in the 3D scaffold GBM cultures lacking a hydrogel and in tumor spheres grown by the traditional serum-free sphere culture methods, respectively. The LDs were found to aggregate mostly extracellularly. The presence of these extracellular LDs may contribute to the observed lower drug sensitivity of specific sub-populations of GBM cells within the silk-HA constructs, consistent with a recent report linking an increase in cytosolic lipids to TMZ resistance in GBM^43^.

To our knowledge, this is the first report to capture the accumulation of LDs with primary GBM cultures in vitro, whereas previously only shown in vivo by magnetic resonance imaging in exclusively high grade GBM as opposed to low grade gliomas or healthy brain tissue^44^. Thus, through the recapitulation of lipid-containing droplets in the 3D brain-ECM-containing microenvironment with patient-derived GBM cells, we provide a robust system to examine pathophysiological behaviors of tumors that could not be captured in previously existing models.

## Discussion

The competition between tumor cell cohesion and interactions with the surrounding ECM is purported to dictate the invasive behavior of brain tumors and their drug sensitivity^45^. In this 3D bioengineered brain tumor model, the silk protein scaffolds infused with ECM hydrogels better represent the in vivo environment by supporting a wide range of tumor phenotypes, i.e., stationary or migratory, as single cells or spheres depending on tumor types/stages, while minimizing model-specific artifacts such as spheres’ aggregation in scaffold-free conventional suspension cultures. Our previous studies utilizing 2D adherent and neurosphere cultures^46^ demonstrated the presence of heterogeneous stem cell populations that contribute to tumor progression^47^. Notably, in these early studies where ependymoma and GBM were used to define conditions for expanding patient cells without losing their stem-like subfractions ex vivo, ECM-dependent performance of patient cells was observed. Expansion as adherent cultures over sphere cultures, particularly on laminin-poly-L-ornithine coated plastic dishes was observed to be more favorable for the growth and maintenance of stem-like cell subfractions ex vivo (Supplementary Fig. 11).

Here, we extended these insights to a 3D bioengineered brain tumor tissue model with a complex ECM microenvironment achieved by the incorporation of decellularized brain-ECM. Whereas authentic human brain-ECM is presently unavailable, we infused native brain-ECM cues in our 3D tissue model by supplementing porcine-derived fetal and adult brain ECMs into a base hydrogel (collagen type I or HA hydrogels). The rationale of choosing a 1:3 ratio of ECM to collagen type I/HA was threefold: to obtain a stable hydrogel that could be maintained in culture, to have a stiffness in the range of native brain (0.1–1 kPa^48^) and to simulate the upregulation of collagen type I and/or HA in the tumor microenvironment, each of which was determined to be approximately 2–3-fold higher in ependymoma/GBM when compared to healthy brain tissue^49,50^ (Supplementary Fig. 12). In doing so, we provide a suitable starting point for addressing the complex tumor cells-brain-ECM interactions, particularly from different developmental stages in the context of tumor progression.

Previously, we have described that rat cortical neuronal cultures resulted into more mature neurons within brain-ECM-containing culture conditions^51^, exhibiting a different response to these decellularized matrices in comparison to the patient tumors as reported here. In the current study, with pediatric ependymoma and adult GBM as examples, we show distinctive growth behaviors of the primary tumor cells in the same 3D ECM microenvironment, suggesting tumor-type-specific interactions with the matrix. Additionally, the cell-ECM interactions involved distinct cell-secreted signals in terms of MMPs/TIMPs, corresponding to each tumor type within the same ECM microenvironment. Patient ependymoma cultures exhibited higher MMP9 release within fetal ECM-enriched constructs (Fig. 5a), correlating with the known prognostic value of MMPs in pediatric ependymomas^31^. On the other hand, upregulated TIMPs (Fig. 5b) with a known MMP activation role, can likely explain why the GBM cells were more migratory in fetal ECM-enriched constructs and also indicate a complex balance between TIMPs/MMPs^34^ leading to morphologically/metabolically heterogeneous populations within GBM cultures^52^.

When combined with transcriptomic profiles of the tumor cells in different microenvironments, these interactions showed similarities that clustered by tumor types as well as ECM conditions. Additionally, many ECM contributors, including CSPGs, were differentially produced depending on the brain tumor type and the culture microenvironment. Furthermore, the highest LDH release in fetal ECM constructs, as well as elevated IL-6 and growth factors are suggestive of necrosis following increased senescence of differentiated populations, which in turn has the potential to render tumor stem-like populations more aggressive via paracrine signaling^52^. Thus, regardless of the tumor type, fetal brain-ECM-containing cultures showed more favorable growth as measured with a wide range of readouts, indicating a complex native brain-ECM-containing microenvironment to be better than base hydrogel alone.

Initiation in fetal ECM-supplemented collagen type I/HA hydrogel appeared to boost the initial in vitro growth of primary tumor cells. Fetal brain-ECM is distinct in composition from the mature adult brain-ECM. Fetal brain-ECM has a higher content of total collagens and GAGs than mature adult brain-ECM (which has higher ratio of sGAGs to collagens); we reported this difference in a previous manuscript^51^. Many collagens (*collagens I, IV, VI*) and GAGs (*CSPG4, HA*) are known to be upregulated in brain tumors in comparison to mature adult brain (Supplementary Figs. 12, 13). GAGs (CSPGs, HSPGs) play an important role in tumor progression, as shown by the work of Phillips and others^7,53^. There are also specific hints in literature towards HA concentration in glioma matching that in embryonic brain tissue^13^ and cancer as an embryological phenomenon^54^, suggesting why the fetal brain-ECM-enriched in collagens and GAGs such as CSPGs, HA^55,56^ best supported tumor growth in vitro in our 3D cultures. The adult brain-ECM, on the other hand, although reported to have a higher ratio of sGAGs to collagens and comparable to mature adult brain, due to the additional presence of GAGs and many other ECM proteins likely still modulates/enhances tumor-ECM interactions than just plain collagen I/HA.

Our study demonstrates the utility of high resolution metabolic imaging to identify morphologically/metabolically distinct tumor cell populations and labile events, such as LDs. The novel finding of GBM cell-produced LDs in the 3D microenvironment can perhaps be attributed to the stabilization of the extracellular droplets by the amphiphilicity of the high molecular weight silk fibroin protein of the scaffold. LD presence is an emerging characteristic of GBM observed in glioma patient samples and correlated with poor survival^57–60^. Although preliminary, as detailed analysis is still needed for validation, this is powerful as it applies to monitoring other tumor types prospectively, perhaps in conjunction with patient chemotherapy in future. While our optical redox ratio assessments can provide highly sensitive and non-invasive assessments of metabolic differences and the spatially heterogeneous responses of cells, there are limitations in terms of the specificity with which these measurements alone can identify the origins of such changes. In the future, optical metabolic imaging could be combined with novel exogenous metabolic activity sensors that may provide such insights^61^. Alternatively, terminal imaging and mass spectrometry measurements may be used to gain more mechanistic insights^62^. Future work would also require laser capture microdissection (LCM) and single cell profiling alongside longitudinal live imaging since many of the contributing factors are temporal.

Our work addresses the complexity of brain tumor-ECM interactions, an active area of research in need of more in-depth knowledge and tools. The compartmentalized design allows for examination of single or combinations of different brain-relevant matrices. Specifically, TPEF imaging combined with bulk solution-based assays allowed for a range of fast readouts. For instance, metabolic imaging results combined with the cytokine profiles pointed towards the critical roles played by both HA and the components in fetal brain-derived ECM for bolstering GBM aggressiveness in vitro, consistent with the known roles of HA in GBM microenvironment^19,63^. This result suggests that we can modulate ECM cues, based on changes during development, in the future to prevent certain tumor cell behaviors. Together, these findings have provided many novel insights to the reciprocal signaling between a brain tumor and its ECM microenvironment that are relevant to understanding the growth and drug sensitivity of 3D tumors. To further increase relevance of these in vitro tumor tissue models to the in vivo environment will need incorporation of non-tumor cells, present in the tumor microenvironment. We tested the feasibility of doing so by combining 3D bioengineered cultures of differentiated neural stem cells with patient tumor cells (Supplementary Fig. 14). One approach to investigate the interactions between the neoplastic and non-neoplastic cells, by exploiting the tunability of these 3D cultures would involve the assembly of two concentric rings of scaffolds seeded with each of the cell types before the introduction of ECM hydrogel.

Feasibility of multiple dosage regimens and repeated manipulation, followed by metabolic imaging for continuous tracking of the same drug treated cultures over time, suggests potential utility of this robust system for drug screening. We acknowledge that we have described this platform using two different primary brain tumors. Considering the low frequency of patients, this made expanding the study to a larger sample size challenging. Our goal was to present the utility of this new tissue model in evaluating patient tumor-ECM interactions. Studies involving more patient tumor samples will be useful to increase confidence in the broader applicability of this new tissue model for drug screening and mechanistic studies. Future studies should involve utilizing this scaffold-based 3D tumor culture platform with multiple fresh samples alongside low passage tumor cells expanded in serum-free media. A personalized approach, as we have demonstrated in this study, can provide a tool to enable more systematic studies of diverse types of brain tumors. Importantly, these studies demonstrate that the tissue model system is highly tunable, amenable to scale-up and easy to adapt to a wide range of biological assays and label-free imaging.

## Materials and methods

### Primary human brain tumor culture

Tumor tissue was harvested during resection surgery after patient consent in accordance with the IRB-12418 protocol, approved by the Institutional Review Boards of Tufts University and Connecticut Children’s Hospital. Following de-identification, each patient tumor sample was transferred in ice-cold DMEM-F12 supplemented with 1% antibiotic-antimycotic (Sigma–Aldrich). Resected tumor tissue was portioned for immediate culturing, and for cryopreserving for future cultures. For viably freezing, each tumor sample was chopped into ~2 mm^2^ pieces and frozen in equal volumes of growth media and fetal bovine serum (FBS) supplemented with 5% dimethyl sulfoxide (DMSO). All the frozen tumor samples were completely used during the course of this study.

Anaplastic ependymoma (Grade III) from a pediatric patient (2-year-old female patient) was mechanically dissociated by sequential pipetting using 10 mL, 5 mL, 2 mL, and 1 mL pipettes until a homogenous cell suspension was obtained. The cell suspension was centrifuged at 1200 r.p.m. for 5 min, followed by resuspension in either FBS free growth media, EGM 2MV-NBM [50% complete neurobasal (NBM) containing base neurobasal with 1% glutamax, 1% B27 supplement, 1% antibiotic-antimycotic; 50% endothelial growth media (EGM 2MV BulletKit minus FBS)] or FBS-containing growth media (DMEM F12 with 10% FBS and 1% antibiotic-antimycotic).

For the adult GBM sample (55-year-old male patient, primary IDH-wild-type, EGFR-positive), the tumor tissue was minced into 1 cm^2^ pieces and incubated in TrypLE Select enzyme (Thermo Fischer Scientific, 12563029) for 15 min at 37 °C, with regular pipetting every 5 min. Upon completion of incubation, twice the volume of GBM growth media was added to the tumor sample. GBM growth media consisted of vitamin-A deprived neurobasal, 1% B27 supplement, 1% antibiotic-antimycotic and the following human growth factors: 20 ng mL^−1^ epidermal growth factor (EGF, Peprotech AF10015100UG), 20 ng mL^−1^ basic fibroblast growth factor (bFGF, vWR 100-18B-100UG), 10 ng mL^−1^ heparin, 20 ng mL^−1^ platelet-derived growth factor-BB (PDGF-BB, Peprotech 100-14B). Next, the minced and digested GBM samples in media were further mechanically dissociated by pipetting using a 1 mL pipette with a broad tip. Since the adult tumor tissue was much more fibrous and stiffer than the pediatric samples, a balance had to be obtained between the pipetting repetitions and complete dissociation of the tumor tissue. Once a relatively homogeneous cell suspension was obtained, it was centrifuged at 1200 rpm for 5 min and aspirated to remove any TrypLE Select enzyme, followed by resuspension in GBM growth media.

Additional steps involved filtering of the adult GBM cell suspension using a 100 µm cell strainer to remove undigested tissue debris. The filtrate was plated on 2D laminin and Matrigel coated plates, as well as grown as spheroids in low adhesion six-well plates. Laminin (Sigma, 11243217001) and Matrigel (Corning, 354277) coatings were prepared by performing dilutions in PBS and DMEM, respectively. The incubation period for the coatings was either overnight at 4 °C or ~2 h at 37 °C. During the passaging of the 2D cultures, TrypLE Select enzyme was used to detach cells and frozen stocks were created from the early passages. For the initial media changes of the spheroid cultures, 40 um cell strainers were used to retain the formed spheroids and the filtrate with single dead cells was discarded. Eventually, to avoid losing spheroids that remained adhered to the cell strainer, media changes for spheroid cultures involved centrifuging the spheroids and resuspending them gently in half fresh media.

### 3D bioengineered brain tissue model with primary tumor cells

The assembly of the bioengineered cortical tissue was performed as previously described with further optimization^64^. Briefly, silk porous 3D scaffolds were coated with 0.1 mg mL^−1^ poly-D-lysine (Sigma–Aldrich) either overnight at 4 °C or for 2 h at 37 °C. The scaffolds were washed with PBS three times and incubated in media at 37 °C for at least 30 min to equilibrate the scaffolds for cell seeding. Subsequently, tumor cells from either the mechanically dissociated pediatric tumor, TrypLE Select digested adult GBM or single cells obtained from primary GBM expanded as spheroids (P2) were seeded in the 3D ring-shaped silk scaffolds. After overnight incubation of 100 µL tumor cell suspension per scaffold in a 96-well plate, the unattached cells were washed away with the growth media for the corresponding tumor type. Next, the tumor cell-seeded scaffolds were infused with 3 mg mL^−1^ rat tail collagen type I (Corning) or HA hydrogels.

For the generation of ECM-collagen type I hydrogels, porcine brain-ECM from different developmental stages was obtained via a previously developed decellularization process^51^. The lyophilized ECM was solubilized with 1 mg mL^−1^ pepsin from porcine gastric mucosa (Sigma–Aldrich) in 0.1 N hydrochloric acid (Sigma–Aldrich). The solubilization time for fetal and adult ECM at room temperature was ~16 and 24 h, respectively. Once solubilized, the ECM was mixed with 10x DMEM (final 1x in gel) and neutralized using 1 M NaOH (Sigma–Aldrich). The neutralized ECM solution was mixed with 3 mg mL^−1^ rat tail collagen type I (Corning) for a final ECM concentration of 1000 μg mL^−1^ and the gelation process started using NaOH. The ECM-collagen solution was kept on ice until complete gelation was required and was used within 2 h of preparation. For HA hydrogels, 5.5% tyramine-substituted HA (Lifecore) was reconstituted sterile at 10 mg mL^−1^ in ultrapure water overnight at 4 °C on a shaker. To obtain HA gels of ~ 1kPa bulk modulus, final optimized concentrations of HA (4 mg mL^^−1^), horseradish peroxidase (1 U mL^−1^), hydrogen peroxide (0.005% v/v) and pH adjusted 10x DMEM (1x in gel) were mixed together on ice. The remaining volume was adjusted by addition of ultrapure water. In the case of ECM-HA hydrogels, 10x DMEM (with a final concentration of 1x in gel) was added to solubilized ECM, which was further pH adjusted and then mixed with the rest of the components. HA hydrogels were prepared in small volumes (~1 mL) due to their rapid gelation time and added to scaffolds immediately. After introduction within the cell-seeded scaffolds, the gelation was completed within 30 min at 37 °C, following which more media was added to each well with the constructs. The next day, each of the ECM-containing tumor cell-seeded 3D constructs was moved to a larger well of a 24-well plate with sufficient media.

We tested the response of the 3D tumor cultures to the chemotherapy drugs that were administered to the patients, cisplatin (Sigma–Aldrich, 232120) and TMZ (Selleckchem, S1237) for anaplastic ependymoma and GBM, respectively. Anaplastic ependymoma samples were treated at 4 mo with multiple doses (2–3) of cisplatin over a period of 2 weeks; while GBM samples were treated at ~7mo in culture with a single dose of 200 µM TMZ for 72 h. We started with TMZ concentrations based on published in vitro concentrations, which were tested mostly in 2D glioblastoma cell lines^65^. We performed in vitro screening to check for TMZ concentration in 3D cultures that reduced viability, such that we could image the changes by metabolic imaging. Control samples were exposed to DMSO in media for the same time frame as drug treated samples.

### Co-culture with differentiated healthy hiNSCs

3D bioengineered constructs with healthy differentiated induced neural stem cells (hiNSCs) were generated using a similar methodology as described for tumor cultures and previously detailed for hiNSCs in the silk-scaffold system^66^, with further optimization. The differentiating hiNSC constructs were transfected with a viral vector, AAVdj-hSyn-eYFP (Charu Ramakrishnan, Karl Deisseroth, Stanford University), such that the differentiated synapsin (Syn)-expressing neurons would express eYFP (Yellow fluorescent protein). GBM cells expanded as spheres from the patient-tumor were placed in close proximity to the 3D hiNSC constructs 7 mo post-differentiation. Before starting the co-cultures, the GBM cells were separately incubated with 5-aminolevulinic acid (5-ALA, Sigma–Aldrich A3785) for 48 h, for visualization following 5-ALA preferential accumulation. The invasion of healthy hiNSC cultures by the GBM cells and vice versa was observed 48 h post co-culture, by simultaneous live imaging of the differentiated hiNSCs by eYFP and GBM by 5-ALA.

### Viability assay

Cell proliferation was performed via WST-1 assay (Sigma–Aldrich) according to the instructions provided by the manufacturer, to assess tumor cell proliferation. Briefly, the samples were incubated for 1 h with WST-1 reagent diluted 1:10 (v:v) in culture medium, followed by a reading of the medium absorbance with plate reader (Molecular Devices) at 450 and 600 nm. Fresh medium was used as a baseline control and its average absorbance was subtracted from the value of the samples. A total of *N* = 3−6 samples/condition were used in the assay depending on the tumor type.

### Lactate dehydrogenase assay

Lactate dehydrogenase (LDH) released into media by ruptured tumor cells was used as a measure of cell death at different time points during the 3D culture without having to sacrifice the samples. LDH assay was performed according to the manufacturer instructions (Sigma–Aldrich). Briefly, culture medium was mixed with the assay reagents in a 1:2 ratio. Following 30 min incubation at room temperature, the reaction was stopped by addition of 1 N HCl. The absorbance was measured at 490 nm and 690 nm. Once again, fresh medium without any construct was used as a baseline control and its average was subtracted from the sample values. A total of *N* = 3−6 samples/condition were used in the assay depending on the tumor type.

### CSPG release ELISA

CSPGs released by the tumor cells in media were measured using an ELISA based assay^67^. Media samples from the 3D tumor constructs were incubated overnight at 4 °C in a 96-well immuno plate (Thermo Fischer Scientific). Alongside the sample media incubation, chicken extracellular CSPGs (Millipore, CC117) were used over a range of serial dilutions for the generation of standard curves. Following washes with PBS-Tween, monoclonal anti-chondroitin sulfate antibody produced in mouse/clone CS-56, ascites fluid (Sigma–Aldrich, C8035) was added for overnight incubation at 4 °C. After the next round of washes, HRP conjugated goat anti-mouse secondary antibody (Abcam) was incubated at room temperature for 2 h. TMB (3,3′,5,5′-tetramethylbenzidine) 1-C Substrate (Fischer Scientific) was introduced following the last round of washes with PBS-Tween. Finally, after the color developed for 10 min at room temperature, the reaction was stopped with 1 N HCl. The absorbance readings were measured at 450 nm wavelength and the fresh media readings were subtracted from the sample readings. The standard curve was utilized for calculating the quantities of CSPGs released in the different conditions and reported in pg mL^−1^.

### Immunostaining

The samples were fixed at different time points with 4% paraformaldehyde (PFA) solution in PBS (Santa Cruz Biotechnology). Fixation time was 10 min for 2D samples and 20–30 min for the 3D constructs. The cells were stained with glial fibrillary acidic protein (GFAP)primary antibody (Sigma G3893) at a 1:500 dilution and incubations were performed at 4 °C overnight, while the secondary antibody (Invitrogen A-11001, 1:250 dilution) incubations were carried out at room temperature for 2 h. The stained samples were imaged using the Leica SP8 confocal microscope with ×10 or ×25 objectives.

### Live mitochondrial imaging

2D ependymoma cells or 3D ependymoma constructs were incubated in media containing diluted TMRE (30 nM) (Thermo Fischer Scientific) and Mitotracker (300 nM) (Thermo Fischer Scientific) at 37 °C for 10 min and 1 h, respectively. Following incubation, the samples were imaged using the Keyence BZ-X700 or the Leica SP8 confocal equipped with an incubator setup maintained at 37 °C and with 5% carbon dioxide inflow. The images were taken with ×60 objective using the same exposure time, and light power across all sample conditions when using the Keyence BZ-X700 for the 2D samples.

### 5-ALA imaging

During long-term culture (4 mo) of GBM cells, tumor cell invasion in different matrices was examined via migration into the ECM filled central window of the cell-seeded ring-shaped scaffold via 5-ALA (Sigma–Aldrich A3785) accumulation within tumor cells. 5-ALA was excited at 405 nm and emission was collected within 620–720 nm, corresponding to the protoporphyrin IX which accumulates in glioma cells due to low ferrochelatase activity. The 3D samples were imaged using the Leica SP8 confocal microscope.

### RNA sequencing and qRT-PCR

Samples were flash frozen in liquid nitrogen and stored in −80 ^o^C in individual RNAase free eppendorf tubes until RNA extraction was performed. All samples were placed on dry ice during extraction, sequentially disrupted using a liquid nitrogen chilled bio-pulverizer. Between each sample, the pulverizer was wiped with 70% ethanol to remove remnants of the previous sample, and between each sample set (different conditions), all the tools were cleaned with RNAzap. Lysis buffer was immediately added to the powdered frozen sample and placed on ice. Once all the samples were in lysis buffer on ice, a 22-gauge needle and syringe were used for sample homogenization one by one using a fresh needle and syringe every time. All the samples were spun down to remove undigested material (mainly silk scaffold) and the supernatant was transferred to clean RNAase free eppendorfs. Following this, the SurePrep All Prep kit (Fischer Scientific) protocol was followed until RNA was eluted from the columns. Preliminary RNA concentrations were measured using nanodrop (Nanodrop 2000, Thermo Fischer Scientific) and sent to Jackson labs for Genomic Medicine in Connecticut, MA for sequencing. Quality control was performed on the RNA samples post cleanup and DNAase treatment for improved RIN values. Next, a KAPA mRNA stranded library was generated for RNA sequencing (RNA-seq) on the Illumina HiSeq 4000.

The raw sequencing data were evaluated at Tufts Genomic Core where the fastq reads were quality checked, preprocessed and aligned to the reference human genome, Hg38 using HISAT2. The aligned data were mapped to possible transcripts using Cufflinks. The final assembled transcriptome or the output FPKM (Reads Per Kilobase of transcript per Million mapped reads) values were imported in Qlucore Omics Explorer for visualization and plotting.

For qRT-PCR, RT2 First Strand Kit with an incorporated gDNA removal step with buffer GE (Qiagen) was utilized for cDNA synthesis from the eluted RNA. cDNA samples were mixed with RT2 SYBR Green Fluor qPCR Mastermix and added to the Qiagen RT2 PCR Array (including a housekeeping gene, genomic DNA control and RT control). PCR was run on BioRad CFX96.

### Microarrays for MMP/TIMP and cytokine release

Multiplex Quantibody matrix metalloproteinases/tissue inhibitors of matrix metalloproteinases (MMP/TIMP) and cytokine arrays (RayBiotech) were used to semi-quantitatively or qualitatively compare the proteases/protease inhibitors and cytokines released by the tumor cells cultured in the 3D bioengineered brain tumor model with different ECM environments. Small volumes of control media samples/cell culture supernatants (50 µL or 2 mL) from the 3D constructs were incubated in the capture antibody spotted glass slides or the membranes, along with the standards provided that corresponded to known concentrations of the targets for the Quantibody arrays. This overnight incubation was followed by another overnight step at 4 °C, involving the biotinylated detection antibody cocktail. Next, streptavidin-conjugated fluorophore or HRP-streptavidin was added for 1 h at room temperature. Finally, the slide was disassembled from the removable gasket, dried, and scanned using a fluorescence microarray laser scanner (Ray Biotech). For the membrane array, chemiluminescence detection agent was added right before imaging the membrane.

The human MMP array Q1 (Ray Biotech) allowed for simultaneous detection of six MMPs and three TIMPs: MMP-1,2,3,9,10,13, TIMP-1,2,4. Additionally, the human cytokine array C5 (Ray Biotech) was used to determine the release of 80 different growth factors and inflammatory cytokines (Supplementary Table 1). Protein expression profiles of the pediatric and adult brain tumors across the varying ECM conditions were quantified using the Q-analyzer software (Ray Biotech) to obtain the final values in pg mL^−1^ for the Quantibody arrays. For the membrane array, chemiluminescence measurements were quantified using ImageJ and fold change over the control media was reported in heat maps.

### Metabolic imaging of 3D bioengineered brain tumor model

Tissue samples were imaged with a multiphoton confocal microscope (TCS SP8, Leica) equipped with a Ti-Sapphire laser and time-correlated single-photon counting electronics. During imaging, each sample was placed in a well of a glass bottomed (No. 1.5 coverslip) 24-well plate. The imaging chamber was maintained at 37 °C and humidified along with a continuous supply of 5% CO_2_. Endogenous two-photon excited fluorescence (TPEF) images were acquired at 1024 by 1024 pixels with FOV 232.50 µm by 232.50 µm, and 2x zoom using a 25× (0.95 NA) water objective following excitations at 755 nm and 860 nm for capturing NADH and FAD signals, 460 ± 25 nm (NADH) and 525 ± 25 nm (FAD), respectively.

During endogenous imaging, multiple single planes (*n* > 3) at varying depths were acquired within each 3D construct. Power measurements of the laser at the two excitation wavelengths were taken regularly and used to normalize the signals during analysis. Incident laser power was ~20 mW at the acquisition focal plane with a pixel dwell time of 600 ns. CARS imaging targeting the C–H molecular stretch was also performed on a subset of samples to validate lipid content of the droplets observed within the GBM cultures. CARS imaging was performed using a commercial Leica TCS SP8 CARS microscope (Wetzlar, Germany), equipped with a picoEmerald picosecond laser and optical parametric oscillator (Stokes beam = 1064 nm, 7 ps; pump beam = 770–850 nm, 5–6 ps). Images (295 × 295 microns, 1024 × 1024 pixels, scanning speed of 400 Hz) were acquired with the pump wavelength set at 816 nm in order to optimally excite the C–H vibrational lipid stretch at 2856 wavenumbers. The CARS signal was detected at 650 ± 105 nm. A HC PL IRAPO 40 × /1.10 water immersion objective was used and the average power of each beam at the sample was held below 50 mW to avoid photodamage of the sample^68^.

Following the last session of endogenous imaging, some representative samples were co-stained with CellEvent Caspase-3/7 green (Thermo Fischer Scientific), NucBlue Dead (Thermo Fischer Scientific), and TMRE (30 nM) (Thermo Fischer Scientific) reagents to confirm the presence of dead cells and for morphological comparison post-TMZ treatment. Similarly, a few samples were co-stained with a NucBlue Live (Thermo Fischer Scientific), BODIPY 493/503 (2 µg mL^−1^ in PBS) (Thermo Fischer Scientific) and TMRE (30 nM) to test for the presence of lipids within the droplets observed during imaging.

Field based redox ratio calculation (FAD/FAD + NADH), an intensity based-metric, was determined using MATLAB processing routines as described previously^41,69^. In brief, all acquired images were normalized for power and detector gain prior to processing. For a given field, the 755 nm excitation, 460 nm emission (NADH channel) and 860 nm excitation, 525 nm emission (FAD channel) were then spatially co-registered by determining the shift maximizing correlation between the two channels. A mean normalized redox ratio value was computed for each field as the normalized fluorescence intensity contributions from FAD over the sum of the intensity contributions from NADH and FAD and reported as the mean redox value per field of view. Additionally, a lifetime based filter was utilized to remove silk contributions. Briefly, silk autofluorescence has a longer fluorescent lifetime during excitation at 755 nm than that of NADH found in cellular regions. Silk scaffold areas were removed by masking out regions that possessed lifetime values beyond a cutoff threshold value when this lifetime was transformed into phasor space (transforming the exponential decay of fluorescence lifetime with sines and cosines), so that there was minimal loss of cellular signal^23,41,70^. The overall image-based redox ratios were measured after drug treatment with TMZ or control DMSO, and across different ECM conditions. The signals captured at the excitation/emission pair 755/460 ± 25 nm could be the contributions of both NADH and NADPH, as these are indistinguishable from each other. However, we have shown previously NADPH to be present at negligible levels as opposed to NADH in multiple cell types and settings, and thus we assess the optical redox ratio as based on the concentrations of NADH and FAD.

We further proceeded with the determination of redox ratio distribution components using a subset of representative images (*n* = 3–11). Pixel based redox ratio histograms were first generated by applying a spatial Gaussian low-pass filter to each redox map image and then creating 50 bins between redox ratio values 0 and 1. Images from different groups were compiled and used in the gmdistribution.fit function in MATLAB to fit the aggregate histograms with Gaussian components. The basic components for the model were determined as an average of means and standard deviations of 10 iterations. We then fit these basis components to each histogram and normalized relative weights to sum to 100%. To quantify changes in redox histograms between groups, image-wise ratios of the various Gaussian component weights were used (i.e., W1/ (W2 + W3 + W4), W2/ (W1 + W3 + W4), etc.). Component 1 (centered at the lowest redox ratio) is typically associated with glycolysis, while higher components have been paired to enhanced oxidative phosphorylation and oxidative stress conditions.

### Statistics

The graphs were prepared, and statistical analysis performed using GraphPad Prism 7 software (GraphPad, CA, USA). All data are expressed as mean ± s.d arising from sample size of *n* ≥ 3. The analysis methods utilized included one-way ANOVA, followed by Tukey’s post-hoc test to determine the statistically significant differences for multiple comparisons with one independent variable (ECM, media) and unpaired two-tailed *t*-test for comparison of two groups unless stated otherwise in the figure captions. A 95% confidence interval or 0.05 was used as the threshold for significance (*p < 0.05) throughout all the statistical tests. Stars associated with *p*-values have no bearing on the results and conclusion, and are simply reported as obtained from Graphpad prism for the ease of showing individual *p*-values. Appropriate regression curve was used to fit a trendline on the standard curve generated for ELISAs, such that R^2^ < 0.97. During the generation of heat maps with hierarchical clustering and principal component analysis (PCA) plots from RNA-seq data, false discovery rate q < 0.2 or *p*-values < 0.005 were used to identify discriminating variables between comparison groups. Additionally, z-score method, which centers the data to zero mean and unit variance, was used to scale the data presented in heat maps corresponding to RNA-seq data^22^.

## Supplementary information

Supplementary Information

Supplementary Data 1

Supplementary Movie 1

Source Data

## Data Availability

Source data are available for Fig. 2, 57, and Supplementary Figs. 3, 4, 6, 7–9 in the Source Data file. Source data for Figs. 3 and 4 are provided as Supplementary Data 1. The accession code for the RNA-seq data deposited to GEO is GSE134567. Any additional information is available from the authors upon request.
