# Supplementary material for: 3D extracellular matrix microenvironment in bioengineered tissue models of primary pediatric and adult brain tumors
Source: Nat Commun. 2019 Oct 4;10:4529. doi: 10.1038/s41467-019-12420-1 (PMC6778192; doi:10.1038/s41467-019-12420-1)
Supplement: Supplementary file 1 — Supplementary Information [file 41467_2019_12420_MOESM1_ESM.pdf]

3D Extracellular Matrix Microenvironment in Bioengineered Tissue Models of Primary Pediatric and Adult Brain Tumors

Sood et al.

Supplementary Information

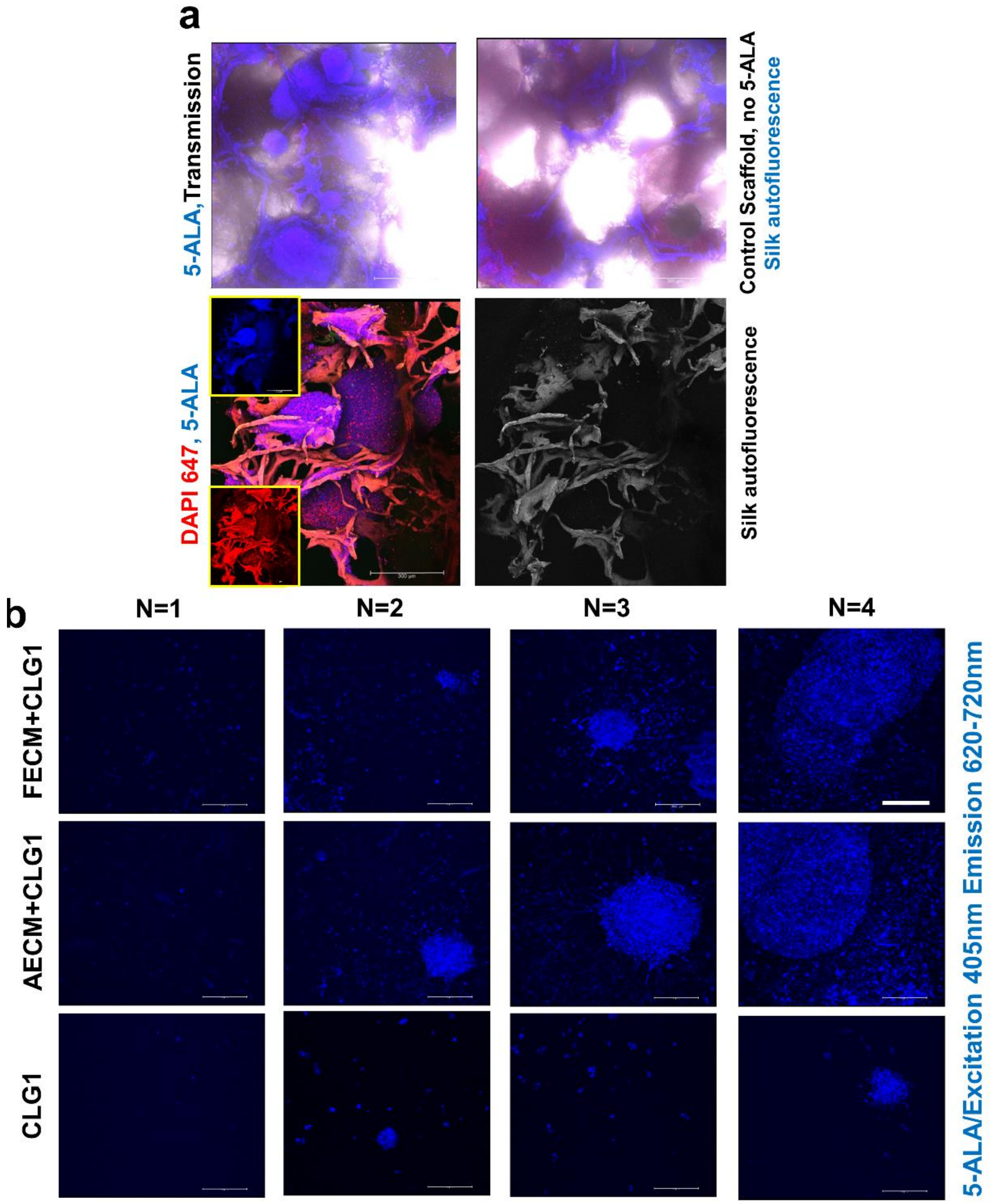

Supplementary Figure 1: 5-aminolevulinic acid live imaging of glioblastoma cultured in 3D constructs for tracking migration into the central hydrogel window (a) 5-Aminoluvelinic acid (5-ALA) preferential accumulation (post 48-90hrs incubation) and fluorescence in 4 mo GBM cells within the 3D silk-scaffold constructs as seen in blue against a background of silk transmission image (top left) and overlay with live DAPI stain (bottom left). Control scaffolds without added 5-ALA and DAPI 647 to indicate silk autofluorescence levels as seen in the corresponding right panels. Max projection of Z-stacks imaged in Leica SP8 confocal. Scale bar 300 μm. (b) Migration of GBM cells within the central hydrogel window at 4 mo across different hydrogel conditions as measured live by 5-ALA. Max projection of Z-stacks imaged in Leica SP8 confocal. Scale bar 100 μm. N refers to separate scaffold samples.

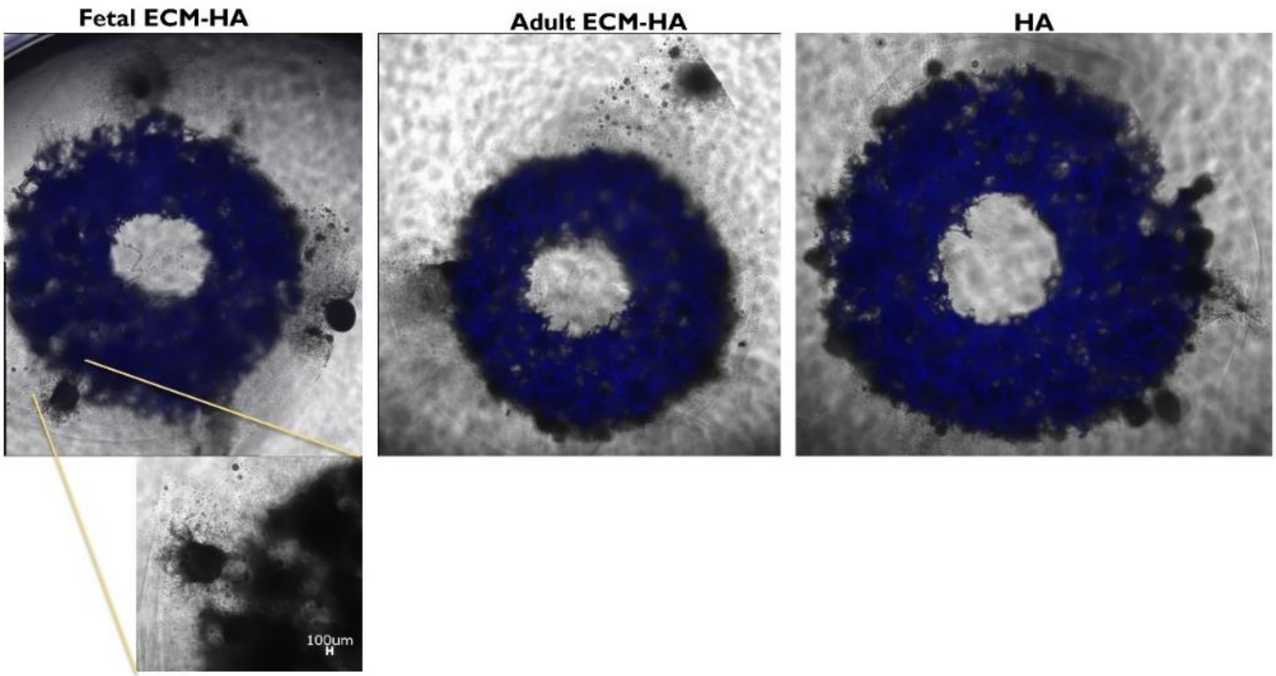

Supplementary Figure 2: GBM cell seeded-scaffold infused with HA-based hydrogels indicating migration of fibrous tumor spheres in the gel extending out from the outer ring of the scaffold referred to as outer edge gel. Greatest migration in the outer edge gel was noticed within fetal-ECM enriched HA cultures as indicated in the left inset. DAPI channel overlaid to demarcate the scaffold using its autofluorescence in this channel. Scale bar=100µm.

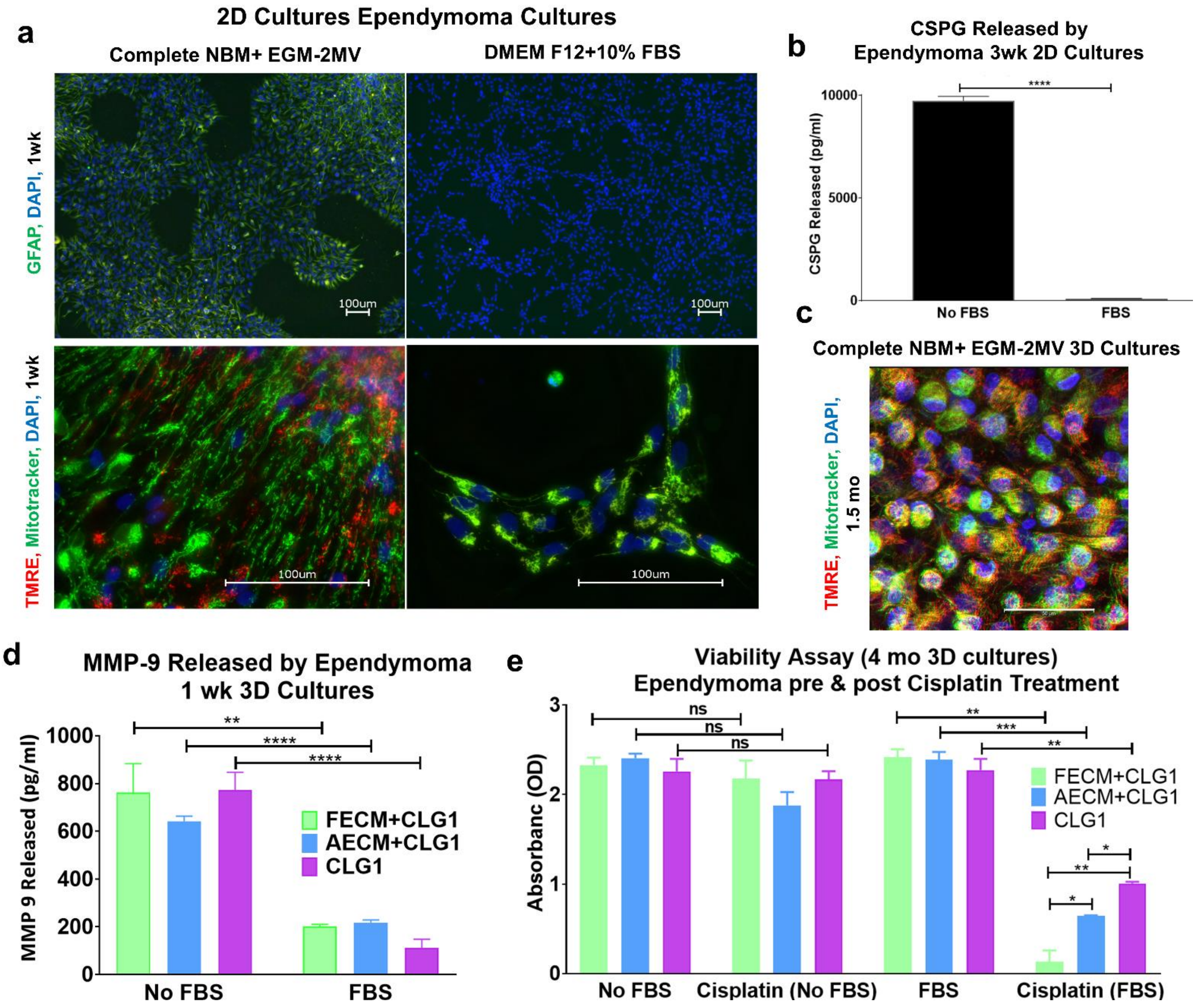

Supplementary Figure 3: Media optimization for culture of patient brain-derived tumors leading to altered drug response. (a) Pseudo-rosettes characteristic of ependymoma observed in tumor cells cultured in 2D in media lacking FBS (complete neurobasal: NBM+ endothelial media EGM-2MV) as shown by GFAP stained cells (upper left panel) and greater capacity of mitochondrial activity was indicated by Mitotracker that stains all mitochondria in conjunction with TMRE staining for mitochondrial membrane potential (lower left panel), as opposed to cells grown in FBS containing DMEM+F12 media (right panel). Scale bar 100µm. (b) Chondroitin sulfate proteoglycan (CSPG) release observed to be significantly higher in 2D cultures of ependymoma in media lacking FBS. Unpaired two-tailed t-test, ****$p<0.0001$. (c) Metabolic activity of ependymoma in 3D constructs at 1.5 mo. High capacity of mitochondrial activity in ependymoma cells within 3D constructs when cultured in serum free media, was indicated by dual mitochondrial staining with MitoTracker and TMRE. Max projection of Z-stacks imaged using Leica SP8 confocal. Scale bar 100µm. (d) MMP-9 release in media from ependymoma 3D cultures at 1 wk significantly greater in media lacking FBS, regardless of the infused hydrogel. Unpaired two-tailed t-test, n=4, **$p=0.0026$, ****$p<0.0001$. (e) Similar viability seen across different ECM and media conditions in 4 mo 3D ependymoma cultures, however the response to cisplatin treatment was more pronounced in the cultures with FBS containing media. Unpaired two-tailed t-test between individual pairs assuming equal SD, n=3 before cisplatin treatment, n=2 post-cisplatin treatment; *$p< 0.0330$, **$p< 0.0046$, ***$p= 0.0008$, ****$p<0.0001$. Error bars indicate mean±s.d. Source data are provided as a Source Data file.

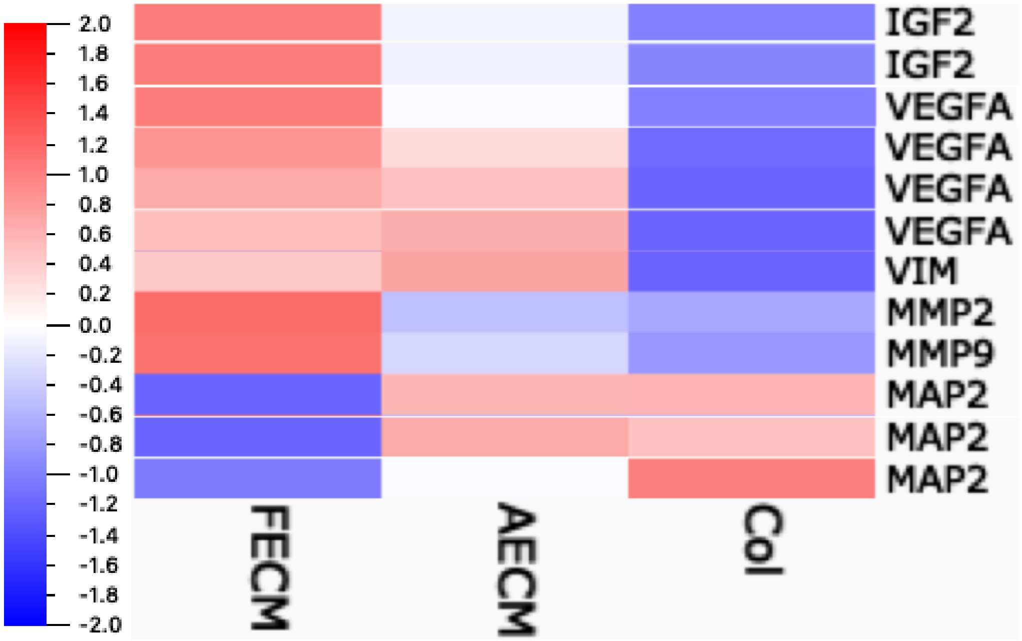

Supplementary Figure 4: Some genes relevant to anaplastic ependymoma from the RNA sequencing data of 3D bioengineered cultures. Heat map generated using FPKM (Reads Per Kilobase of transcript per Million mapped reads) values. Multiple gene variants/isoforms shown for VEGFA: vascular endothelial factor A, MAP2: Microtubule-associated protein 2, IGF2: Insulin growth factor 2. VIM: Vimentin, MMP9: Matrix metalloproteinase 9, MMP2: Matrix metalloproteinase 2. Source data are provided as a Source Data file.

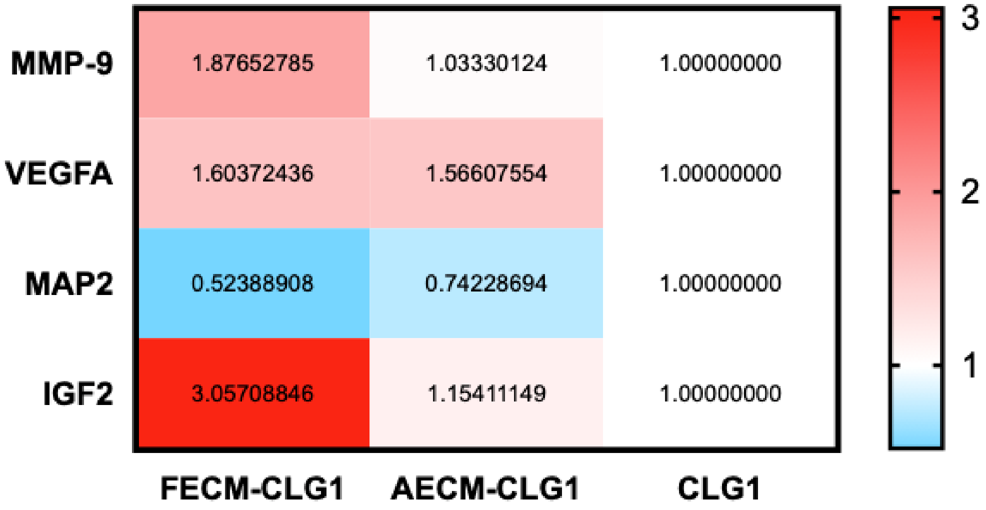

Supplementary Figure 5: mRNA expression of select genes known to be altered in ependymoma, shown here to be altered within ependymoma bioengineered cultures as measured using PCR. Fold expression change relative to unsupplemented collagen (CLG1) cultures.

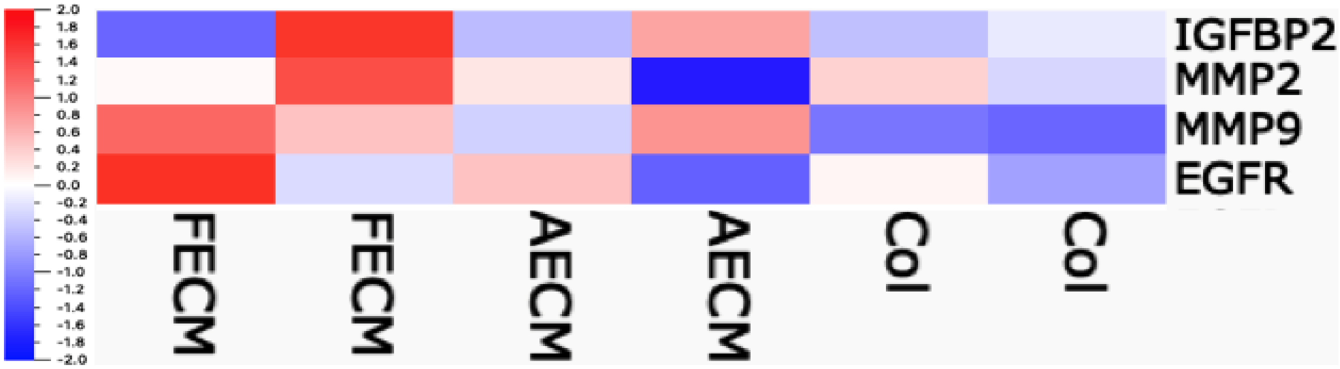

Supplementary Figure 6: Some genes relevant to glioblastoma from the RNA sequencing data of 3D bioengineered cultures. Heat map generated using FPKM (Reads Per Kilobase of transcript per Million mapped reads) values. IGFBP2: Insulin Like Growth Factor Binding Protein 2, MMP2: Matrix metalloproteinase 2, EGFR: Epidermal growth factor receptor, MMP9: Matrix metalloproteinase 9. Source data are provided as a Source Data file.

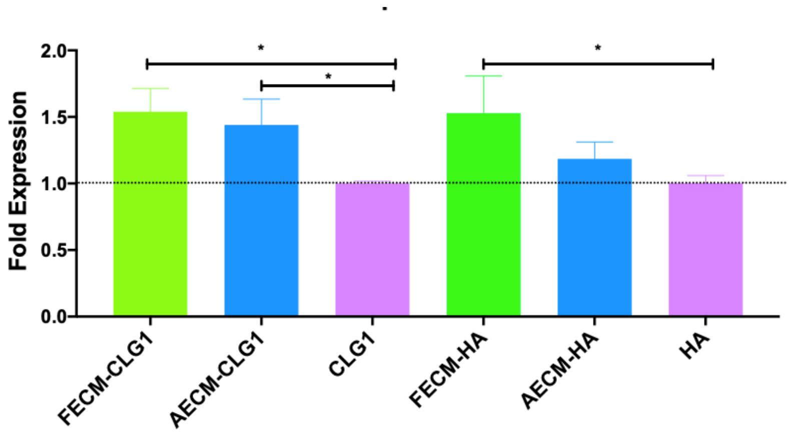

Supplementary Figure 7: EGFR mRNA expression in GBM bioengineered cultures. For the CLG1 (collagen I) set n=2/ condition, for HA (hyaluronic acid) set n=3/ condition. Fold expression relative to CLG1/HA as control conditions. One-tailed t-test to compare to control CLG1/HA condition for the corresponding sets assuming equal SD; *$p < 0.0434$. Error bars indicate mean±s.d. Source data are provided as a Source Data file.

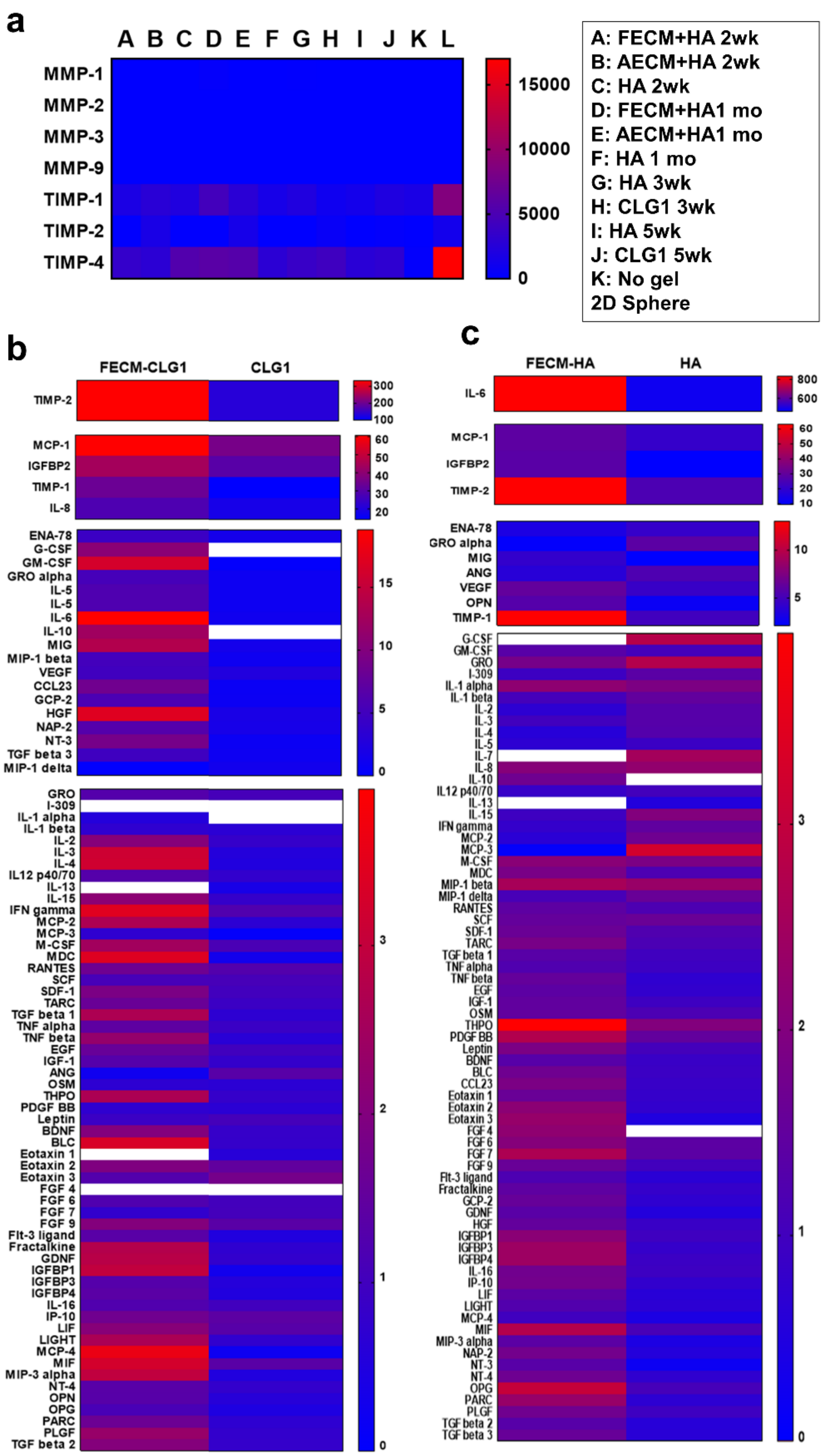

Supplementary Figure 8: Entire panel of cytokine and MMP/TIMP profiling of media from glioblastoma cultures. (a) MMP/TIMP release profile of 3D GBM cultures infused with HA-based hydrogels alongside 3D GBM constructs with no hydrogel added and 2D sphere cultures (reported in pg/ml). (b) Complete cytokine release profile at 1 mo of 3D GBM cultures infused with CLG1-based hydrogels. (c) Cytokine release profile at 6.5 mo of 3D GBM cultures infused with HA-based hydrogels 5.5 mo time point (reported as fold change over control media). Source data are provided as a Source Data file.

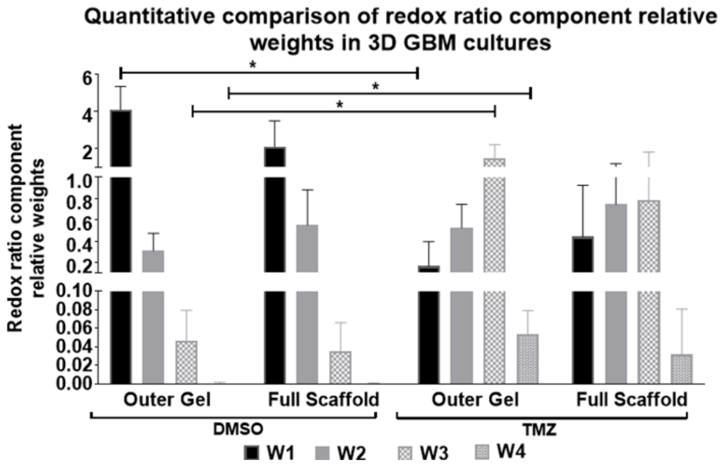

Supplementary Figure 9: Quantitative comparison of redox ratio component relative weights in the 3D GBM cultures. The relative weights in the control DMSO treated samples suggest a highly glycolytic cell population in both groups, as Component 1 is most prominent in the distributions. Following TMZ treatment, Components 3 and 4 become more highly weighted, representative of more oxidative phosphorylation and decreased glycolysis. The outer gel and scaffold groups respond differently to treatment as cells in the outer gel have significantly greater relative weight in both Component 3 and 4, suggesting increased oxidative stress in these cells. “W1” = W1/ (W2 + W3 + W4), “W2” = W2/ (W1 + W3 +W4), “W3” = W3/ (W1 + W2 + W4), “W4” = W4/ (W1 + W2 +W3), etc.). *$p < 0.0347$; Unpaired two-tailed t-tests between separate pairs within each component assuming equal SD; n=3/ condition (multiple areas imaged per condition). Error bars indicate mean±s.e.m. Source data are provided as a Source Data file.

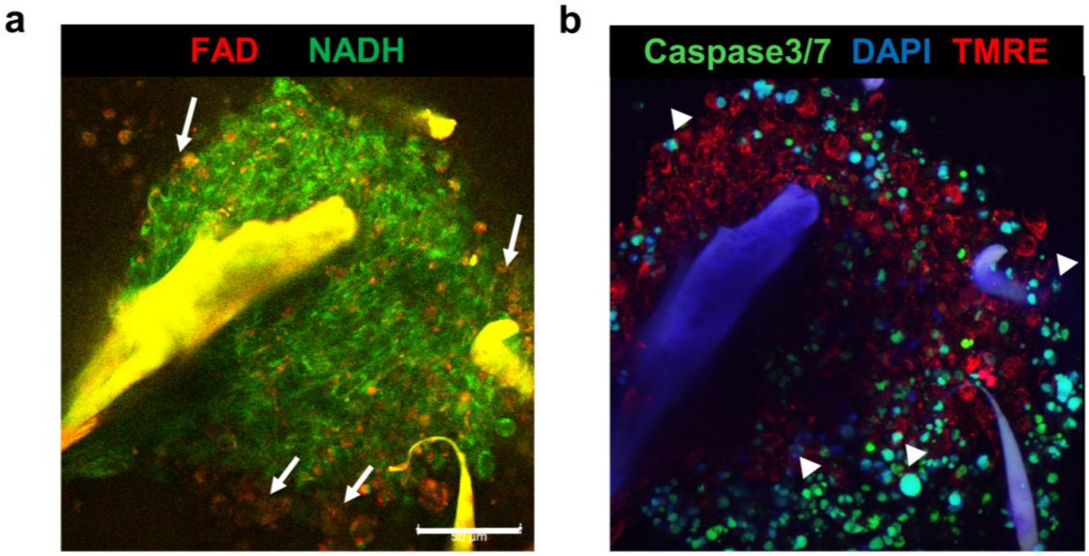

Supplementary Figure 10: Staining of apoptotic cells post metabolic imaging. (a) Overlaid endogenous fluorescence signals of NADH and FAD from GBM cells within silk-HA constructs, imaged by two-photon excitation at 755nm and 860nm, respectively. Cells demonstrating morphological changes shown by arrows. The bright yellow areas correspond to silk. (b) Cells co-stained post endogenous imaging with caspase 3/7 and DAPI indicating apoptotic cells (indicated by arrow heads), imaged using confocal microscope. Dark blue areas correspond to silk. Scale bar 50µm.

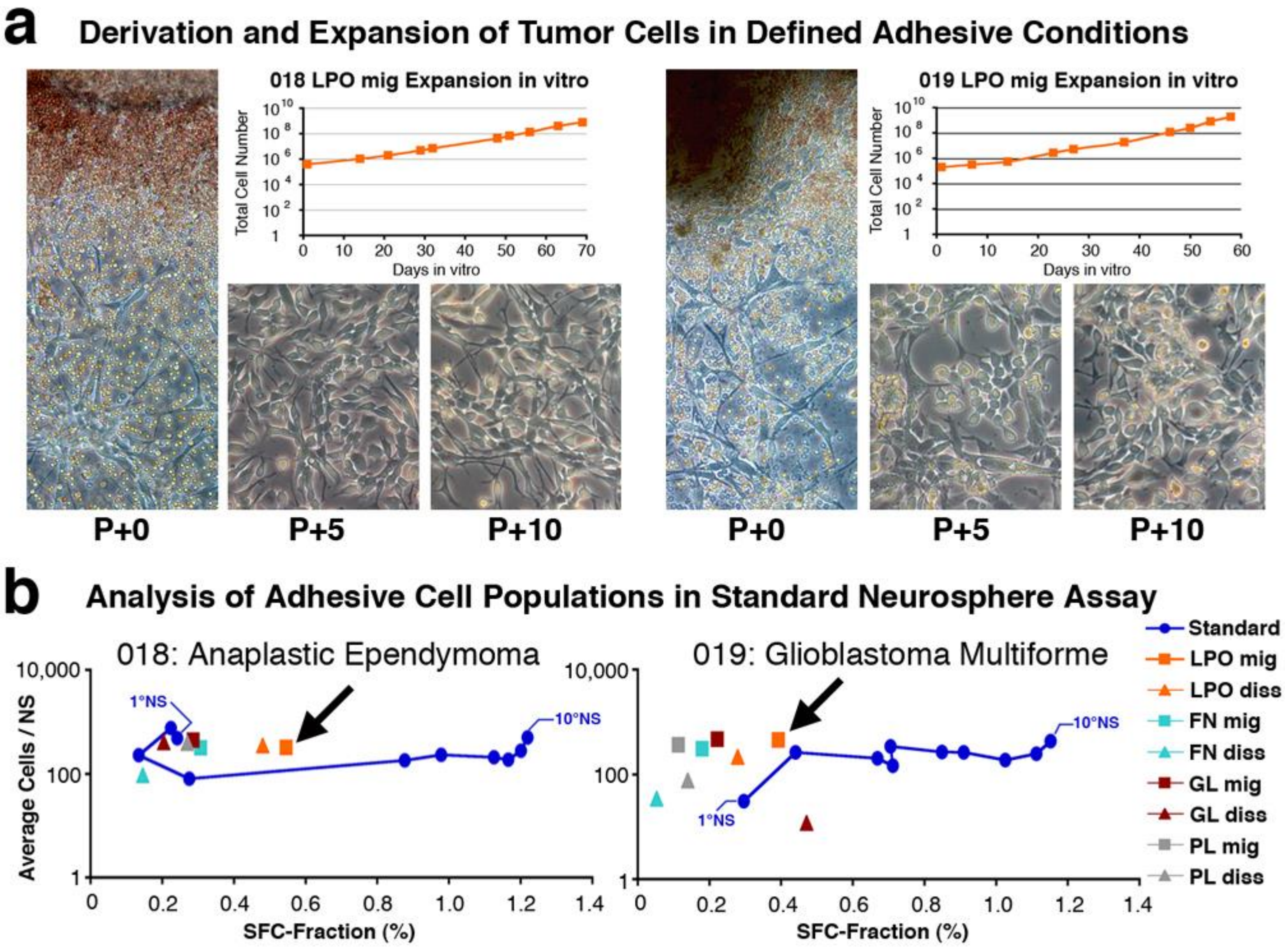

Supplementary Figure 11: Expansion of tumor cells in defined, adhesive conditions to isolate, maintain and expand human brain tumor-derived long-term self-renewing sphere-forming cells (SFCs). (a) Migratory competence and preference to attach to distinct substrates at the time of tissue extraction were compared from the tumor specimens 018-anaplastic ependymoma and 019-glioblastoma *(GBM)*. In this study, a total of eight different cell populations were chosen for further analysis based on their migratory competence and preference to attach to distinct substrates at the time of tissue extraction. In both cases, adhesive cell populations expanded stably over prolonged periods of time (evaluated up to 20 passages corresponding to 25±5 population doublings, PD) without any obvious signs of senescence, or change in morphology. (b) At passage 5 (3-7 PDs), substrate-specific cell populations underwent analysis for SFC presence and activity. Migratory cells (mig) derived on Laminin / poly-L-ornithine-coated culture dishes (LPO mig populations) contained highest numbers of SFCs, outperforming the isolation efficacy of the standard NS assay for both brain tumor specimens. Resident *non-migratory* cells: diss.

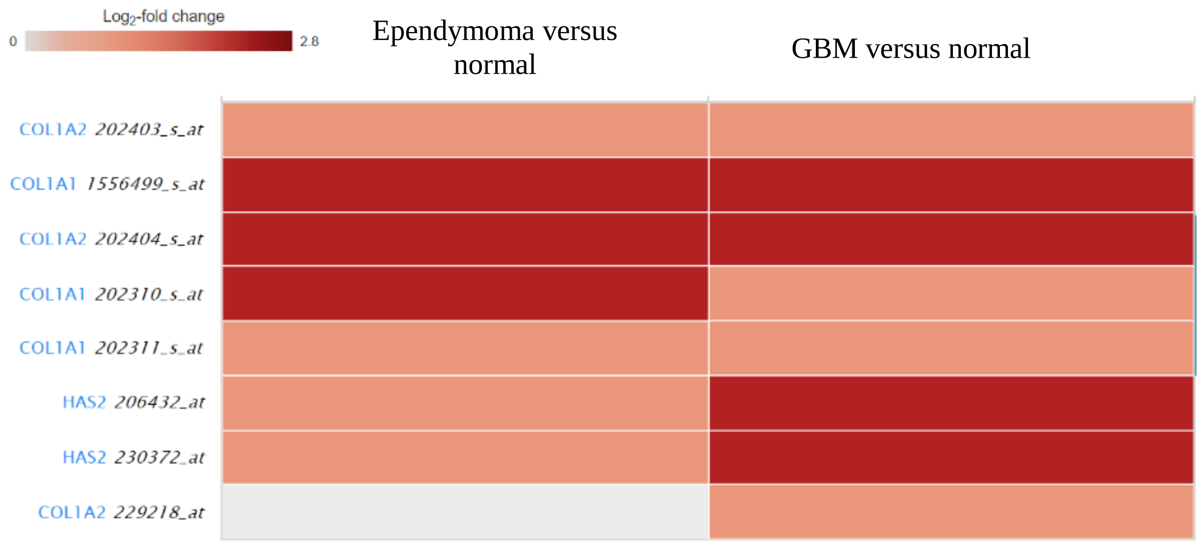

Supplementary Figure 12: Differential mRNA expression data from human brain tumors versus normal human brain indicating the upregulation of different collagen I subtypes and hyaluronic acid synthase 2 transcripts in ependymoma and GBM, measured using Affymetrix HU-133PLUS 2.0 GeneChip microarrays [54, 82]. Log-fold change over normal brain tissue.

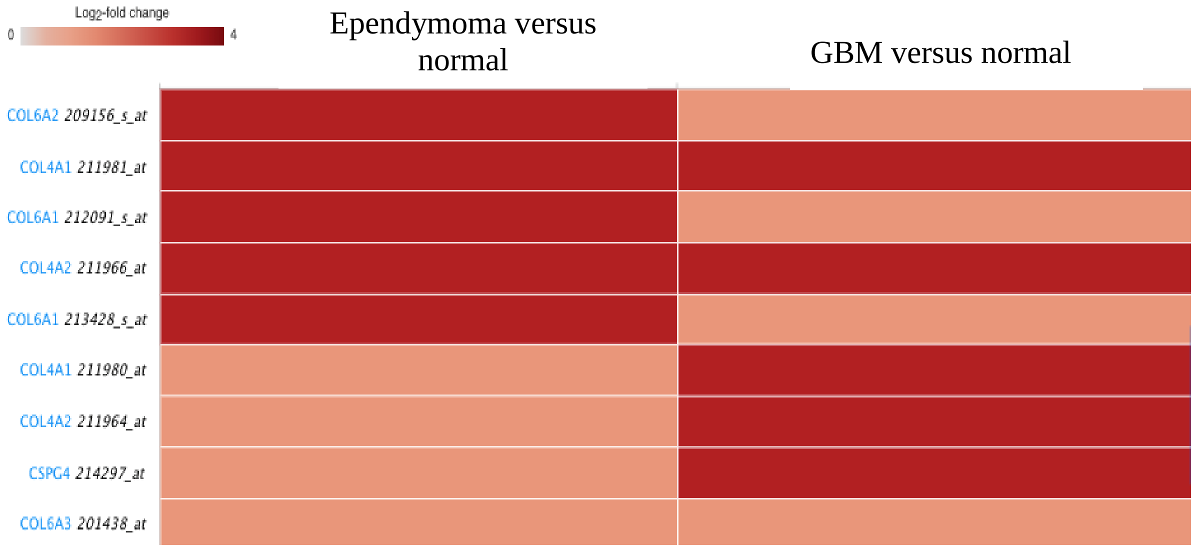

Supplementary Figure 13: Differential mRNA expression data from human brain tumors versus normal human brain indicating the upregulation of many collagens and GAGs in ependymoma and GBM, measured using Affymetrix HU-133PLUS 2.0 GeneChip microarray. Log-fold change over normal brain tissue.

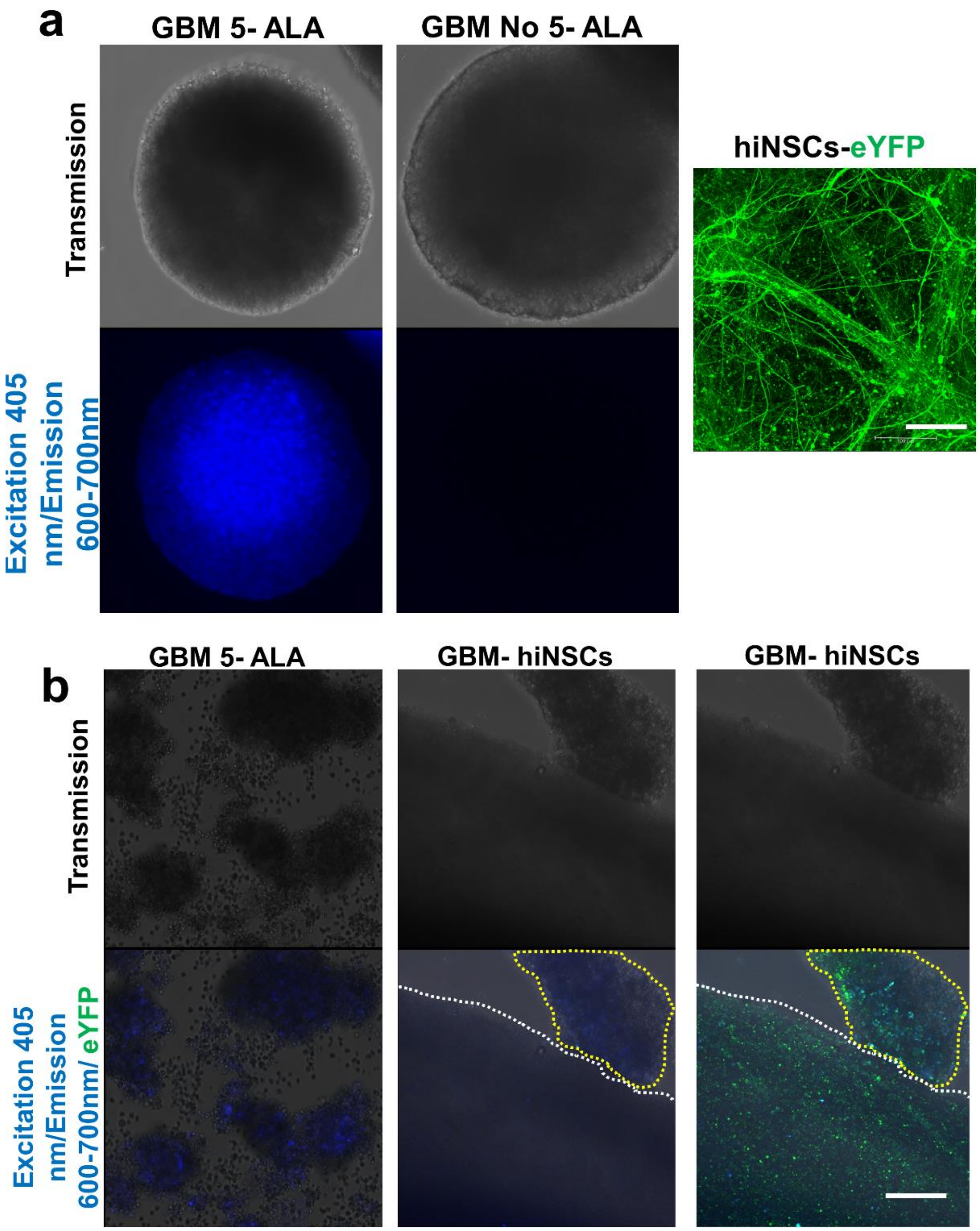

Supplementary Figure 14: Coculture of glioblastoma with differentiated human neural stem cells (hNSCs). (a) GBM spheres before combining with hNSCs, with the left panel indicating 5-ALA stained GBM cells post 24 hrs of 5-ALA addition and middle panel the control GBM cells without 5-ALA addition. Differentiated neurons from hNSCs at 4 mo expressing eYFP (rightmost image). (b) 24 hrs post combining 5-ALA stained GBM cells with e-YFP expressing differentiated hNSCs to obtain co-cultures in complete neurobasal media (neurobasal+ B27+ glutamax). Left panel shows unattached GBM cells. Middle panel indicates 5-ALA stained GBM cells (demarcated in yellow) in close proximity and co-culture with hNSCs (demarcated in white), excitation at 405 nm and imaged at an emission wavelength of 600-700nm. Right panel indicates overlay of GBM cell emission based on 5-ALA with emission from hNSCs based on eYFP, indicating cell infiltration post 48 hrs of co-culture. Scale bar=100µm.

Supplementary Table 1: List of cytokines and matrix metalloproteinases tested in 3D tumor cultures

ANG	Angiogenin
BDNF	Brain-derived neurotrophic factor
CCL1	Chemokine ligand 1/I-309/TCA-3
CCL11	C-C motif chemokine 11/Eotaxin-1
CCL13	Chemokine ligand 13/MCP-4
CCL15	Chemokine ligand 15
CCL17	Chemokine ligand 17/TARC
CCL18	Chemokine ligand 18/PARC
CCL2	Chemokine ligand 2/MCP-1
CCL20	Chemokine ligand 20
CCL22	Chemokine ligand 22/MDC
CCL23	Chemokine ligand 23/CK beta 8-1
CCL24	Chemokine ligand 24/Eotaxin-2
CCL26	Chemokine ligand 26
CCL4	Chemokine ligand 4
CCL5	Chemokine ligand 5/RANTES
CCL7	Chemokine ligand 7/MCP-3/MARC
CCL8	Chemokine ligand 8/MCP-2
CSF1	Colony stimulating factor 1
CSF2	Colony stimulating factor 2
CSF3	Colony stimulating factor 3
CX3CL1	Chemokine (C-X3-C motif) ligand 1/Fractalkine
CXCL1	Chemokine (C-X-C motif) ligand 1/GRO alpha
CXCL10	Chemokine (C-X-C motif) ligand 10
CXCL12	Chemokine (C-X-C motif) ligand 12/SDF-1 alpha
CXCL13	Chemokine (C-X-C motif) ligand 13/BLC
CXCL2	Chemokine (C-X-C motif) ligand 2
CXCL3	Chemokine (C-X-C motif) ligand 3
CXCL5	Chemokine (C-X-C motif) ligand 5/ENA-78
CXCL6	Chemokine (C-X-C motif) ligand 6/GCP-2
CXCL8	Chemokine (C-X-C motif) ligand 8/IL-8
CXCL9	Chemokine (C-X-C motif) ligand 9/MIG
EGF	Epidermal growth factor
FGF4	Fibroblast growth factor 4
FGF6	Fibroblast growth factor 6
FGF7	Fibroblast growth factor 7
FGF9	Fibroblast growth factor 9
FLT3LG	Fms-related tyrosine kinase 3 ligand
GDNF	Glial derived neurotrophic factor
HGF	hepatocyte growth factor
IFNG	Interferon gamma (IFNγ)

IGF1	Insulin-like growth factor 1
IGFBP1	Insulin-like growth factor-binding protein 1
IGFBP2	Insulin-like growth factor-binding protein 2
IGFBP3	Insulin-like growth factor-binding protein 3
IGFBP4	Insulin-like growth factor-binding protein 4
IL10	Interleukin 10
IL12A	Interleukin 12 A
IL13	Interleukin 13
IL15	Interleukin 15
IL16	Interleukin 16
IL1A	Interleukin 1A
IL1B	Interleukin 1B
IL2	Interleukin 2
IL3	Interleukin 3
IL4	Interleukin 4
IL5	Interleukin 5
IL6	Interleukin 6
IL7	Interleukin 7
KITLG	Kit ligand/Mast cell growth factor MCF/Stem cell factor SCF
LEP	Leptin
LIF	Leukemia inhibitory factor
LTA	Lymphotoxin-A
MIF	Macrophage Migration Inhibitory Factor
NTF3	Neurotrophin 3
NTF4	Neurotrophin 4
OSM	Oncostatin M
PDGFB	Platelet-derived growth factor subunit B
PGF	Placental growth factor
PPBP	Pro-platelet basic protein
SPP1	Osteopontin
TGFB1	Transforming growth factor beta-1 (TGFβ-1)
TGFB2	Transforming growth factor beta-2 (TGFβ-2)
TGFB3	Transforming growth factor beta-3 (TGFβ-3)
THPO	Thrombopoietin
TNF	Tumor necrosis factor
TNFRSF11B	Osteoprotegerin
TNFSF14	Tumor necrosis factor superfamily member 14/LIGHT
TPO	Thrombopoietin
VEGFA	Vascular endothelial growth factor A
MMP1	Matrix metalloproteinase1
MMP10	Matrix metalloproteinase10
MMP13	Matrix metalloproteinase13
MMP2	Matrix metalloproteinase2

MMP3	Matrix metalloproteinase3
MMP8	Matrix metalloproteinase8
MMP9	Matrix metalloproteinase9
TIMP1	Tissue inhibitor of matrix metalloproteinase1
TIMP2	Tissue inhibitor of matrix metalloproteinase2
TIMP4	Tissue inhibitor of matrix metalloproteinase4
